# Experimental and theoretical investigations on the high-electron donor character of pyrido-annelated N-heterocyclic carbenes

**DOI:** 10.3762/bjoc.12.178

**Published:** 2016-08-23

**Authors:** Michael Nonnenmacher, Dominik M Buck, Doris Kunz

**Affiliations:** 1 Organisch-Chemisches Institut, Ruprecht-Karls Universität Heidelberg, Im Neuenheimer Feld 250, D-69120 Heidelberg, Germany; 2 Institut für Anorganische Chemie, Eberhard Karls Universität Tübingen, Auf der Morgenstelle 18, D-72076 Tübingen, Germany (current address of corresponding author)

**Keywords:** carbonyl complexes, electron donor character, N-heterocyclic carbene, rhodium

## Abstract

Rh(CO)_2_Cl(NHC) complexes of dipyrido-annelated N-heterocyclic carbenes were prepared. From the C–H coupling constant of the respective imidazolium salts and the N–C–N angle of the N-heterocyclic carbene (NHC), a weaker σ-donor character than that of typical unsaturated NHCs is expected. However, the IR stretching frequencies of their Rh(CO)_2_Cl complexes suggest an electron-donor character even stronger than that of saturated NHCs. We ascribe this to the extremely weak π-acceptor character of the dipyrido-annelated NHCs caused by the conjugated 14 πe^−^ system that thus allows for an enhanced Rh–CO backbonding. This extremely low π-acceptor ability is also corroborated by the ^77^Se NMR chemical shift of −55.8 ppm for the respective selenourea, the lowest value ever measured for imidazole derived selenoureas. DFT-calculations of the free carbene confirm the low σ-donor character by the fact that the σ-orbital of the carbene is the HOMO−1 that lies 0.58 eV below the HOMO which is located at the π-system. Natural population analysis reveals the lowest occupation of the p_π_-orbital for the saturated carbene carbon atom and the highest for the pyrido-annelated carbene. Going from the free carbene to the Rh(CO)_2_Cl(NHC) complexes, the increase in occupancy of the complete π-system of the carbene ligand upon coordination is lowest for the pyrido-annelated carbene and highest for the saturated carbene.

## Introduction

N-Heterocyclic carbenes form a ligand class that is typically characterized by a strong σ-donor and a weak or even negligible π-acceptor effect [1–3], although Meyer has shown pronounced π-acceptor ability in Cu complexes [4–6]. In recent years many varieties of N-heterocyclic carbenes have been synthesized [7–8], focusing mainly on a strong σ-donor character, for example by increasing the ring-size [9–15], substituting one nitrogen atom by carbon [16–17] or using diamido backbones [18–19] and only rarely on enhancing the π-donor character by using π-electron donating backbones [20]. Many efforts have been made to determine and compare the donor abilities of N-heterocyclic carbenes including DFT calculations [2–6,21–24], among which are the most prominent examples: the Tolman-parameter [25–28], the ^13^C NMR chemical shift of special Pd(NHC)_2_ complexes [29–30], and electrochemical properties [31–33] (see [34–35] for reviews). In all these cases, only the overall donor-abilities of the NHC ligand are obtained. In the case of the Tolman parameter, not only the electronic properties of the carbene influence the CO stretching modes, but also steric effects and the coupling of stretching modes. The latter two drawbacks have recently been overcome by calculating the metal–ligand electronic parameter (MLEP) [36]. Separating the influence of the σ-donor and π-acceptor abilities was limited to determining the overall donor character and taking into account the σ-donor character. The latter is dependant on the s-character of the σ-orbital and thus can be obtained directly from the ^1^*J*_CH_ coupling constant of the imidazolium salt [37–39] (which can be regarded as the H^+^ complex of the carbene and therefore π-influences are avoided) or by the N–C–N angle at the carbene [40], which also correlates with the ^13^C NMR chemical shift [41]. In 2013, Ganter presented the ^77^Se NMR chemical shift of the respective selenoureas as a suitable probe to determine directly the π-influence of the carbene [42–43], as the paramagnetic shift tensor has the largest influence on the ^77^Se NMR chemical shift. This method is so far redundant [44] to the method of determining the ^31^P NMR chemical shifts of the respective NHC–phosphinidene adducts [45–46].

**Figure 1:**
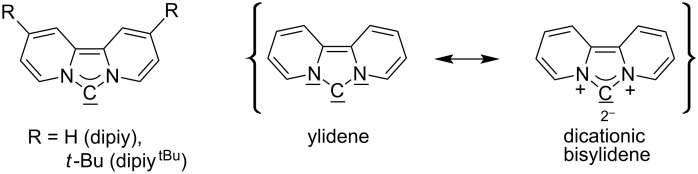
Left: resonance hybrid of the dipyrido carbenes dipiy and dipiy^tBu^. Right: two canonical forms of the dipyridocarbene according to Weiss and co-workers.

For some years we have worked with pyrido-annelated N-heterocyclic carbenes, an NHC class that was introduced by Weiss and co-workers (Figure 1) [39,47]. They pointed out the unusual high s-character of that carbene σ-orbital by a ^1^*J*_CH_ coupling constant of 232.6 Hz, which corresponds to a hybridization of only sp^1.15^. Although they had prepared the respective selenourea, the ^77^Se NMR chemical shift was not reported [39,47–48]. We showed that the *tert*-butyl substituted dipyridocarbene dipiy^tBu^ exhibits an unusual high thermal stability and proofed the alternating bond lengths in the conjugated π-system of this carbene (similar to heptafulvalene) as well as the very low N–C–N angle by X-ray structure analysis [41]. Weiss proposed this carbene to have a “built-in umpolung” [39] ability which means that there could be a participation of the dicationic bisylidene resonance form as it is usually described for carbodiphosphoranes [49] and carbodicarbenes [50–51], in which the carbon atom has a formal oxidation state of ±0 (Figure 1) [52–54]. Earlier, we had prepared their tungsten and chromium carbonyl complexes, but could not find deviations of the CO stretching frequencies from those of analogous NHC complexes [55]. As this might be due to the distribution of the effect on five carbonyl ligands, we now prepared the [Rh(CO)_2_Cl(dipiy)] complex to obtain a more sensitive probe. In the following we will provide the experimental evidence that dipyrido-annelated carbenes are indeed not only weak σ-donors but also the weakest π-accepting carbenes derived from imidazole so far. This overcompensates even the lower σ-donor character, so that their overall electron-donating ability lies in between that of acyclic diaminocarbenes and saturated NHCs.

## Results and Discussion

### Synthesis of the rhodium CO and ^13^CO complexes **2a** and **2b**

**Scheme 1:**
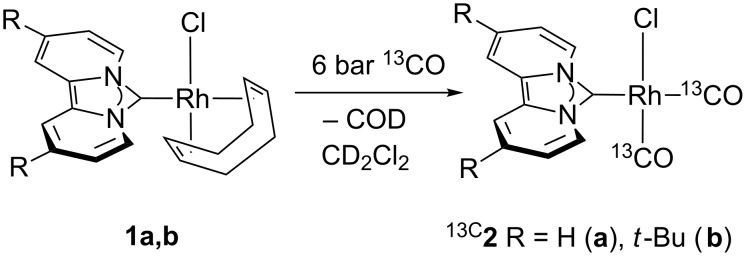
Preparation of the ^13^CO substituted rhodium complexes **2** bearing the dipyrido-annelated carbenes dipiy (**a**) and dipiy^tBu^ (**b**).

We generated the desired carbonyl complexes **2a** and **2b** from the respective COD complex **1** [56] by ligand exchange under a ^13^CO atmosphere of 6 bar in CD_2_Cl_2_ in a pressure-NMR tube according to Scheme 1.

**Figure 2:**
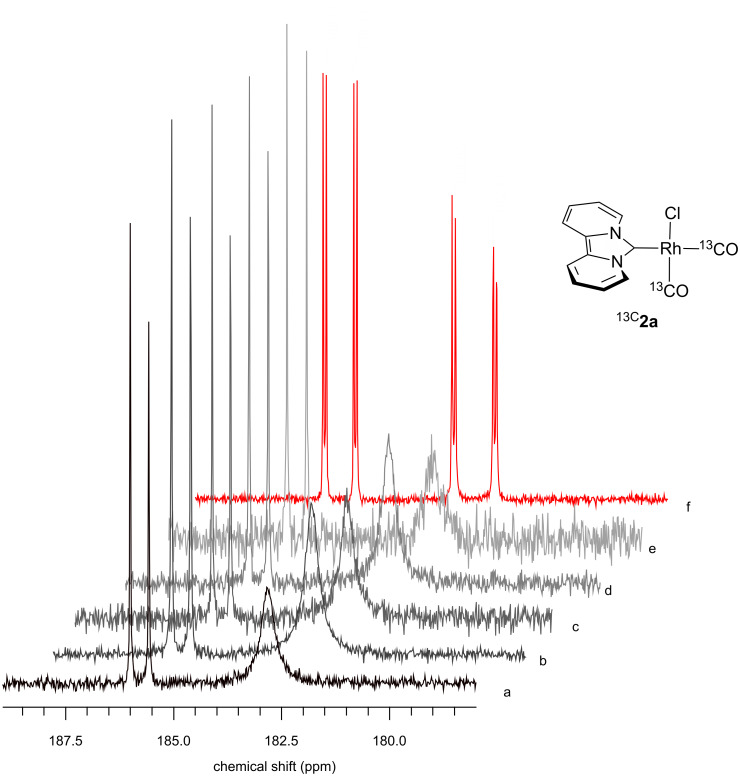
^13^C NMR spectra (carbonyl region, 125 MHz) of the reaction of **1a** with ^13^CO under variable pressure and temperature: left: a) 6 bar ^13^CO, −30 °C; b) 6 bar ^13^CO, −50 °C; c) 1 bar ^13^CO, −30 °C; d) 1 bar ^13^CO, −50 °C; e) grey: 1 bar N_2_ and residual ^13^CO, rt; f) red: ^13^CO-free solution, rt, 75 MHz.

At first, a precipitate forms which is redissolved shortly after and a color change of the solution from light yellow to greenish yellow occurs. The ^1^H NMR spectrum confirms full conversion and release of the COD ligand. Due to a fast exchange with the ^13^CO atmosphere, the respective ^13^C NMR carbonyl signals were not detected. Therefore, we measured the ^13^C{^1^H} NMR spectra at −30 °C. The doublet at 185.9 ppm with a ^1^*J*_RhC_ coupling constant of 53.6 Hz refers to one ^13^CO ligand, while a broad peak at 183.0 ppm indicates fast exchange of the second ^13^CO ligand with non-coordinated ^13^CO (Figure 2). Neither cooling down the sample to −50 °C nor the release of the ^13^CO pressure to 1 bar changed the spectrum qualitatively. The sample was then shaken in an open atmosphere of nitrogen to remove the non-coordinated ^13^CO. This led to a substantial decrease of the intensity of the broad peak, but only after three freeze-pump-thaw cycles to fully remove residual ^13^CO the former broad signal turned into a sharp doublet of doublets at 183.2 ppm with a ^1^*J*_RhC_ coupling of 72.8 Hz and a ^2^*J*_CC_ coupling to the second ^13^CO ligand of 6.1 Hz. Consequently, the former doublet at 186.3 ppm for the second ^13^CO ligand appears now as a doublet of doublets (^1^*J*_RhC_ = 54.5 Hz and ^2^*J*_CC_ = 6.1 Hz).

A comparison of the ^1^*J*_RhC_ coupling constants with those of *cis*- and *trans*-CO Rh-NHC complexes bearing an additional P donor reveals a smaller ^1^*J*_RhC_ coupling constant for the *trans*-CO ligand and a larger coupling constant for the *cis*-CO ligand (relative to the NHC ligand) [57]. This trend is also observed for the ^1^*J*_RhC_ coupling constants of carbonyl complexes with phosphine ligands [58–59]. Consequently, the signal at 186.3 ppm can be assigned to the *trans*-CO ligand and that at 183.2 ppm to the *cis*-CO ligand (relative to NHC). Thus, it is the *cis*-CO ligand that undergoes a fast CO exchange. The same dynamic behavior is observed for complex ^13C^**2b** containing the *tert*-butyl substituted dipyridocarbene ligand dipiy^tBu^. For iridium complexes [Ir(CO)_2_Cl(NHC)] (NHC = imidazolidin-2-ylidene) a preferred *cis*-CO exchange was reported and an activation energy of 12.7–12.9 kcal/mol was determined by NMR spectroscopy for this process [60]. However, an exchange of the CO ligand by phosphines in [M(CO)_2_Cl(NHC)] complexes (M = Rh, Ir) or even by DMSO [60–62] occurs at the *trans*-CO ligand. In some cases, loss of CO upon formation of dimers can be observed for rhodium NHC complexes [63–65].

**Scheme 2:**
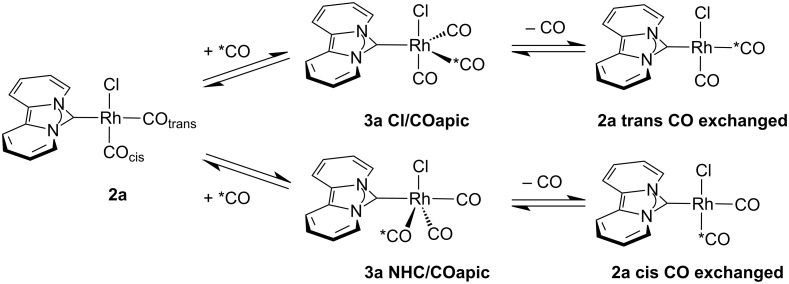
Proposed mechanism for the preferred exchange of the *cis*-CO ligand based on DFT-calculations (BP86 / def2-TZVP) with the dipiy ligand. Intermediate **3a NHC/COapic** is lower in energy by 16.9 kJ/mol compared to intermediate **3a Cl/COapic**.

Ligand exchange in square planar Rh(I) carbonyl complexes was shown to occur by an associative mechanism via a trigonal bipyramidal intermediate [66–67], which was also crystallographically characterized in the case of a cationic Rh complex bearing a bidentate phosphine ligand [68]. Our DFT-calculations for the tricarbonyl complex **3a** bearing the dipiy ligand show that the pentacoordinated intermediate **3a NHC/COapic,** in which the NHC and the former *trans*-CO ligand take in the apical positions, is energetically favored over that with Cl^−^ and the former *cis*-CO ligand in the apical positions (**3a Cl/COapic**) by 16.9 kJ/mol (Scheme 2). The calculated data is similar for complexes with the unsaturated NHC ligand **III**, which favors the respective NHC/COapic intermediate by 14.9 kJ/mol. Assuming similar low activation barriers for the CO association and the dissociation, release of the CO ligand from the trigonal plane in the intermediate **3a NHC/COapic** leads to the preferred exchange of the *cis*-CO ligand (Scheme 2). This is in accordance with the experimental observation of the ^13^CO exchange. Although the formation of **3a NHC/COapic** from **2a** and CO is exothermic (Δ*H*_298K,1bar_ = −11.4 kJ/mol), considering the entropy leads to an endergonic reaction (Δ*G*_298K,1bar_ = 29.4 kJ/mol), even at −50 °C and 6 bar (Δ*G*_223K,6bar_ = 15.7 kJ/mol).

On a preparative scale, complexes **2a** and **2b** were synthesized in a glass autoclave with a CO pressure of 8 bar. In both cases the carbene ^13^C NMR signals (−30 °C, CD_2_Cl_2_) could be detected at 152.2 ppm (^1^*J*_RhC_ = 43.6 Hz) (**2a**) and at 150.3 ppm (^1^*J*_RhC_ = 44.4 Hz) (**2b**). The carbonyl signals are detected at 182.5 (^1^*J*_RhC_ = 72.7 Hz; *cis*-CO) and 185.9 ppm (^1^*J*_RhC_ = 54.0 Hz; *trans*-CO) for complex **2a** and at 182.8 (^1^*J*_RhC_ = 75.8 Hz; *cis*-CO) and 186.1 ppm (^1^*J*_RhC_ = 53.6 Hz; *trans*-CO) for complex **2b**.

To compare the IR stretching frequencies with other Rh-complexes in literature, we determined the symmetric and asymmetric CO stretching modes of complex **2a** in dichloromethane (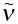 = 2082 and 2003 cm^−1^), dimethyl sulfoxide (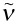 = 2064 and 1984 cm^−1^) and as a KBr pellet (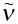 = 2073 and 1993 cm^−1^). This large medium dependence shows that it is mandatory to compare the stretching frequencies analyzed in the same medium. As the difference of the symmetric and the asymmetric CO stretching frequencies is not constant, it is common to compare the average value of these two bands. Table 1 gives an overview of the CO stretching frequencies of [Rh(CO)_2_Cl(L)] complexes with the most common types of NHC ligands L.

**Table 1:** IR carbonyl stretching frequencies of [Rh(CO)_2_Cl(L)] complexes bearing various diaminocarbenes (L).

L in [Rh(CO)_2_Cl(L)]	L	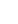 cm^−1^	*ν_av_* cm^−1^ Ø	method

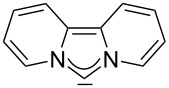	dipiy [39]	2072.8 1993.2	2033.0	KBr, rt
		2082.1 2003.1	2042.6	CH_2_Cl_2_, rt
		2064 1984	2024	DMSO, rt
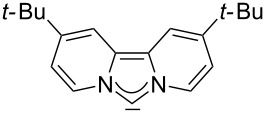	dipiy^tBu^ [41]	2074.5 1996.6	2035.6	KBr, rt
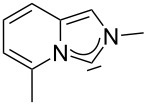	**V** [69]	2079.0 2000.0	2039.5	CH_2_Cl_2_, rt
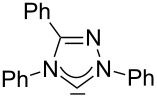	**IV** [70]	2089.0 2009.0 [71]	2049.0	KBr, rt
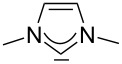	**III** [72]	2076 2006 [73]	2041.0	KBr, rt
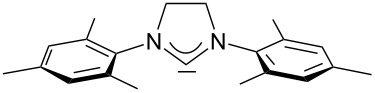	**IIa** [74]	2081.0 1997.0 [75]	2039.0	KBr, rt
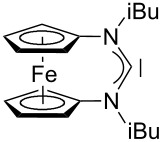	**Ib** [14]	2072 1994	2033	KBr, rt
		2075 1995	2035	CH_2_Cl_2_, rt
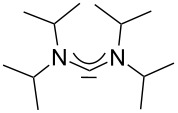	**Ia** [76]	2056.0 1985.0 [75]	2020.5	KBr, rt

**Figure 3:**
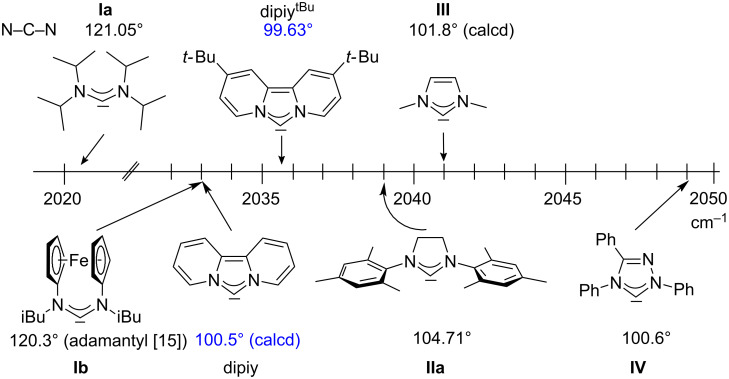
IR scale (cm^−1^) to determine the overall electron-donor capacity of various N-heterocyclic carbenes based on the average wavenumber 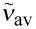 of the CO stretching frequencies of their respective Rh(CO)_2_Cl complexes. The strongest donating NHCs are found on the left side (lower 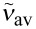), the weakest on the right side (higher 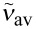). In addition the N–C–N angles are given (from X-ray, if not marked otherwise. For **Ib** the mean value for the *N*-adamantyl substituted species is given).

A graphical illustration of these values is depicted in Figure 3. It shows that the dipyrido-annelated carbenes have an overall donor capacity that lies in between that of acyclic (**Ia**) or ferrocene bridged (**Ib**) diaminocarbenes and saturated imidazolidin-2-ylidenes (**II**). This is surprising, as the σ-donor character of dipyridocarbenes is lower than that of the unsaturated imidazolin-2-ylidenes (**III**) and triazolinylidenes (**IV**), as it can be derived from the low N–C–N angle (99.6°) which enhances the s-character of the carbene σ-orbital and thus reduces the σ-donor character (deduced directly from the larger ^1^*J*_CH_ coupling constant of the respective imidazolium salts that correlates with the higher s-character in the C–H bond). Therefore, we expected the average CO stretching frequencies to lie about 15 cm^−1^ higher at around 2050 cm^−1^ for complexes **2a** and **2b**.

This discrepancy can be explained by a substantial lower π-acceptor character than the generally low π-acceptor character of carbenes. This may even be considered as a π-donor character – as proposed by Weiss and co-workers – to overcompensate the reduced σ-donor property. The description of Weiss and co-workers that dipyridocarbenes had a structural relationship “with “true” bis(ylides) such as carbodiphosphoranes” [39] illustrates very nicely the experimentally determined high overall donor effect of the dipyridocarbene reported herein.

The reason for this behavior seems to be the cross-conjugated 14 πe^−^ system into which the “empty” p_π_-orbital of the carbene is embedded and therefore, could also act as a π-electron donor. To obtain further experimental evidence for this unusually weak π-acceptor (or already weak π-donor character), we determined the ^77^Se NMR chemical shift of the respective selenourea **4b** to be −55.8 ppm [77]. This value is the most negative reported so far for imidazole derived selenoureas and therefore, is another hint for the unusually low π-accepting quality of the dipyridocarbene family dipiy.

To rationalize these strong overall donating properties we performed DFT calculations of the free dipyridocarbene dipiy and its Rh(CO)_2_Cl complex **2a**, as well as the acyclic (**I**, **I-Rh**, **Ia**, **Ia-Rh**), saturated (**II**, **II-Rh**) and unsaturated (**III**, **III-Rh**) diaminocarbenes and their rhodium complexes.

Firstly, we could confirm the trend of the IR stretching frequencies for the calculated complexes although the differences are smaller between **Ia-Rh, 2a**, **II-Rh** and **III-Rh** (Table 2) than observed experimentally. The complex with the isopropyl acyclic carbene **Ia-Rh** shows its unique electron donating effect also in the calculations. The smaller differences found for the calculated CO stretching frequencies are independent of the used functionals (BP86 and B3LYP). Both functionals lead to comparable results with respect to the experimental values when calibrated to free CO (2125 cm^−1^ (BP86), 2208 cm^−1^ (B3LYP), exp. 2143 cm^−1^).

**Table 2:** Calculated values 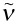 for the symmetric and asymmetric CO stretching frequencies as well as the average 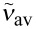 of various Rh(CO)_2_Cl(Carbene) complexes (BP86/def2-TZVP or B3LYP/def2-TZVP) and numbering scheme for the DFT-calculations of the respective carbene.

L	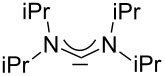 **Ia**	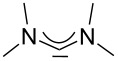 **I**	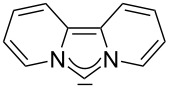 dipiy	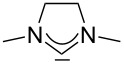 **II**	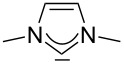 **III**
Rh(CO)_2_Cl(L)	**Ia-Rh**	**I-Rh**	**2a**	**II-Rh**	**III-Rh**

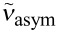 /cm^−1^ 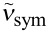 /cm^−1^ (BP86)	1972.4 2047.3	1979.7 2052.6	1983.8 2055.4	1984.1 2056.5	1984.1 2056.8
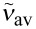 /cm^−1^ (BP86)	2009.8	2016.1	2019.6	2020.3	2020.5
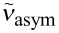 /cm^−1^ 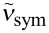 /cm^−1^ (B3LYP)	–	2057.2 2138.9	2062.4 2141.5	2062.2 2142.5	2062.3 2143.2
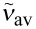 /cm^−1^ (B3LYP)	–	2098.0	2102.0	2102.4	2102.7

To obtain information to which extent the carbene ligands act as an overall π-electron acceptor (or π-donor), we analyzed the electron occupation of the respective p_π_-orbital at the carbene performing a natural population analysis [78] (Table 3). As expected the stronger stabilization of the p_π_ orbital within the conjugated 14 πe^−^ system results in a higher occupation for the carbene dipiy (0.738) at C3, which decreases in the order 6 πe^−^ carbene **III** (0.687), 4 πe^−^ carbene **II** (0.592) and the acyclic 4πe^−^ carbene **I** (0.618) (Table 3). For the rhodium complexes these values are higher, so that an overall π-electron-withdrawing character of the carbene ligand can be concluded. It is surprising that the largest increase in electron occupancy (ΔRh-NHC e^−^ (C3)) is found for the dipyridocarbene dipiy (0.16) and the weakest for the acyclic carbene (**I**) (0.12). However, the increase must not necessarily stem from electron density of the metal center. It could also originate from the π-system of the respective carbene. Therefore, we also calculated the sum of the p_π_-electron occupancy for the free carbene as well as for the complex (which should sum up to 4 e^−^ (**I** and **II**), 6 e^−^ (**III**) or 14 e^−^ (dipiy), respectively). It now becomes clear that the overall gain in π-electron occupancy Δ_Rh-NHC_ e^−^ is highest in the case of the saturated carbene **II** (0.08) and smallest for the dipyridocarbene dipiy (0.04). It may therefore be concluded that dipiy is the carbene with the weakest π-acceptor character. In addition, the role of a potential net π-donor character of the dipyridocarbene dipiy can be ruled out in the Rh complex **2a**. A less electron-rich metal center might induce a net π-donor property in this carbene.

**Table 3:** Electron occupation (e^−^) of the p_π_ orbital at the carbene atom C3 and at the other atoms of the π-system in the free carbenes **I–III** and dipiy and in their Rh carbonyl complexes.

	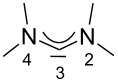 **I**		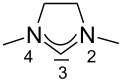 **II**		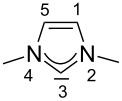 **III**		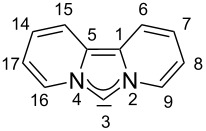 dipiy	
	**I**	**I-Rh**	**II**	**II-Rh**	**III**	**III-Rh**	dipiy	**2a**

C1					1.09029	1.07851	1.08021	1.07529
N2	1.63207	1.60438	1.64093	1.61073	1.54167	1.51623	1.47370	1.45994

C3	0.61791	0.73749	0.59200	0.72812	0.68712	0.82047	0.73731	0.89882

N4	1.63228	1.60384	1.64069	1.61126	1.54169	1.51738	1.47369	1.46334
C5					1.09032	1.07994	1.08021	1.08121
C6							1.00124	1.00100
C7							1.03216	1.00947
C8							1.03375	1.01227
C9							0.98590	0.97578
C14							1.03216	1.01769
C15							1.00123	0.99519
C16							0.98589	0.99538
C17							1.03375	1.01041

Σ πe^−^	3.88226	3.94571	3.87362	3.95011	5.95109	6.01253	13.95120	13.99579

Δ_Rh-NHC_ e^−^ (C3)		0.11958		0.13612		0.13335		0.16151
Δ_Rh-NHC_ e^−^ (π)		0.06345		0.07649		0.06144		0.04459

**Figure 4:**
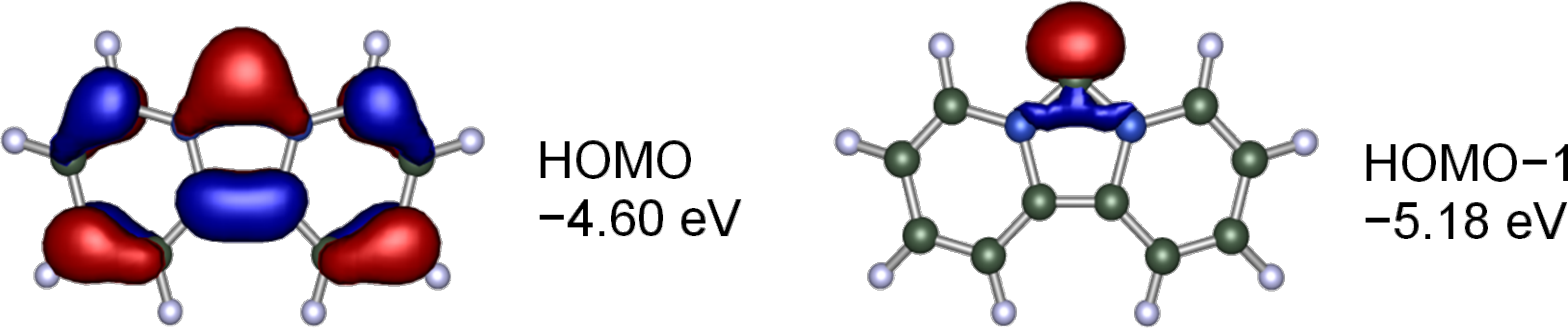
Highest occupied molecular orbitals for the dipyrido-annelated carbene dipiy. The σ-type carbene lone pair is not the HOMO but the HOMO−1.

It is known from theoretical studies that a reduction of the N–C–N angle leads to a stabilization of the carbene σ-orbital [40]. At the same time the extended conjugated π-system leads to an energy increase of the highest occupied π-orbital and a smaller HOMO–LUMO gap. Analyzing the molecular orbitals of the free carbenes **I**–**III** and dipiy reveals that in dipiy the carbene σ-orbital is no longer the highest occupied orbital, but it is found stabilized by 0.58 eV as the HOMO−1 (−5.18 eV). The HOMO at −4.60 eV is located at the π-system (see Figure 4). This has only been observed for the bisoxazoline-derived IBioxMe4 carbene before, whose calculated N–C–N angle of 98.6° is even more acute than that of dipiy [79]. For dipiy the other 6 occupied MOs of the 14 πe^−^ system are found between −6.28 eV and −11.7 eV (HOMO−2 to HOMO−5, HOMO−10 and HOMO−14, see Supporting Information File 1 for a graphical comparison of the highest occupied MOs of **I–III** and dipiy).

The energy gain (Δ*E*_σ_) of the σ-orbitals upon coordination to the rhodium fragment is by far highest for the acyclic carbene **I** (4.14 eV) and lowest for the dipyridocarbene carbene dipiy (3.30 eV) (**II**: 3.57 eV; **III**: 3.66 eV) which displays the order of decreasing σ-donor character (and the decreasing N–C–N angle) of these carbenes.

**Figure 5:**
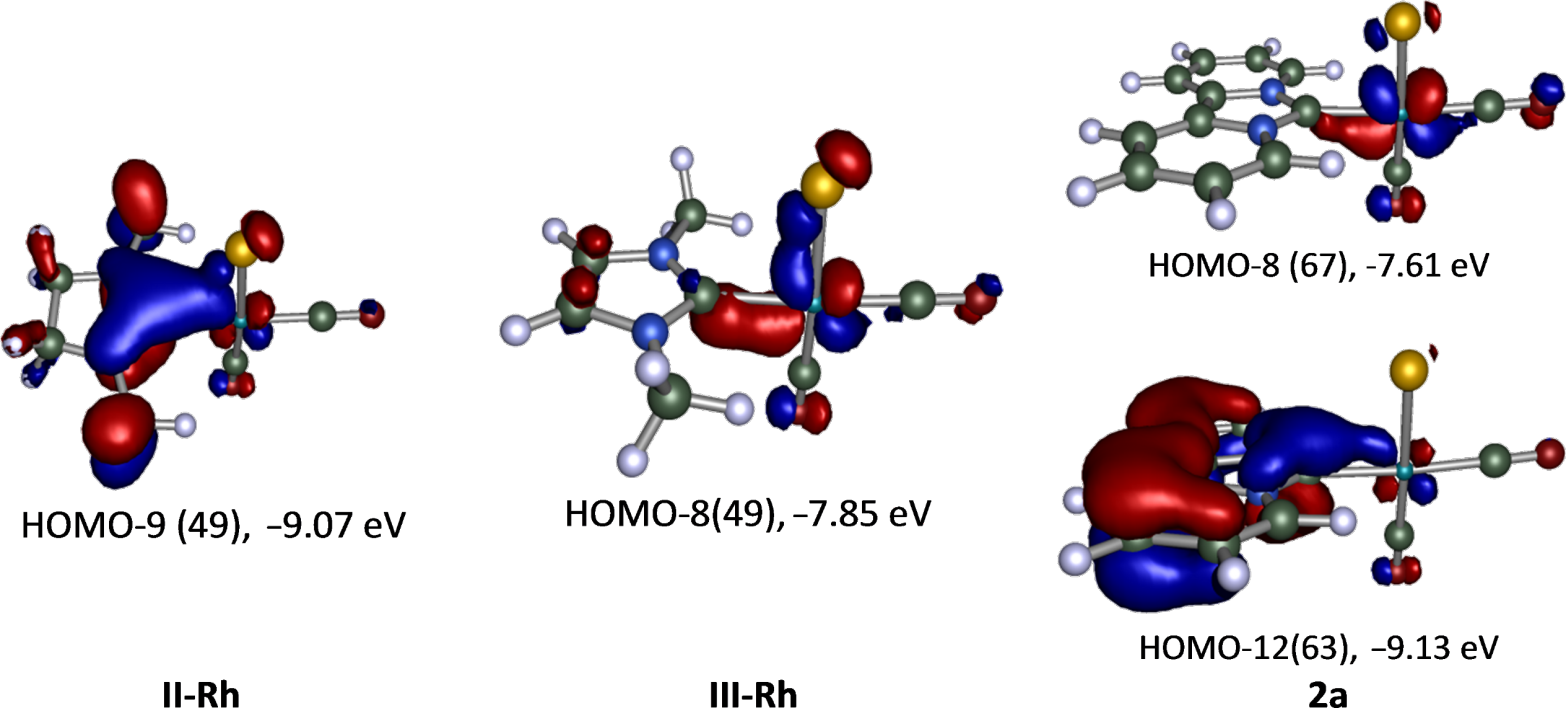
Molecular orbitals of the Rh complexes **II-Rh**, **III-Rh** and **2a** that show ligand-metal π-bonds.

For the Rh-complexes **I-Rh**–**III-Rh** two type of orbitals that indicate a ligand to metal π-donor bond are revealed. One is found for the **II-Rh** complex between the HOMO−2 of the ligand and the d_xy_ orbital of Rh (plus contributions of the chlorido and the antibonding π-orbital of the *cis*-CO ligand) at −9.07 eV (HOMO−9) (Figure 5). The other is found between the HOMO−1 of the carbene ligand and the d_xy_ orbital of Rh in complex **III-Rh** (plus contributions of the chlorido and the antibonding π-orbital of the *cis*- and *trans*-CO ligands) at −7.85 eV. In the case of the dipiy ligand both of these orbital types are recognized at −7.61 eV (HOMO−8) and −9.13 eV (HOMO−12). Tentatively, this could indicate an overall stronger π-donor contribution of this ligand. A molecular orbital that shows an in plane metal-to-ligand π-interaction with the carbene σ*(C–N) orbitals [79] was not observed.

### Explaining the observed CO stretching frequencies

The CO stretching frequencies of imidazolium-derived carbene complexes of Rh are influenced by the sum of σ and π-donor as well as the π-acceptor character of the carbenes. In acyclic diaminocarbene complexes of type **I** the strong σ-donor character dominates as evidenced by the large N–C–N angles and the high lying σ-orbital (HOMO). In the case of complex **Ia-Rh** bearing the isopropyl substituted carbene **Ia**, the steric hindrance of the *N*-isopropyl substituents causes an even larger N–C–N angle. This leads to a further reduction of the s-character and thus an increased σ-donor character that explains the pronounced shift to smaller wavenumbers. According to our DFT calculations, the saturated and unsaturated carbenes have an almost similar donor character so that the weaker σ-donor character is almost compensated by a reduced π-acceptor character. In complexes **2a** and **2b** bearing the pyrido-annelated carbenes dipiy and dipiy^tBu^, the extremely weak π-acceptor character overcompensates the weaker σ-donor effect, so that the overall donor property of the carbene increases and results in CO stretching frequencies that lie in between those of the acyclic (**I-Rh**) and the saturated carbene complex (**II-Rh**).

## Conclusion

We prepared and analyzed both experimentally and theoretically the dipyridocarbene rhodium carbonyl complexes **2a** and **2b**. We showed that the CO exchange of the *cis*-CO ligand is enhanced over that of the *trans*-CO ligand. The unusually high overall donating property of the dipyridocarbenes dipiy exceeds that of imidazolidinylidenes (**II**) and was revealed by IR spectroscopy. The unusually weak π-acceptor character of the dipyridocarbenes was evidenced by the so far lowest ^77^Se NMR chemical shift for imidazole-derived carbenes. Comparing the electron occupancy in the π-system of the free and the coordinated carbene revealed still a, although very low, net π-acceptor character of the dipyridocarbene. We are convinced that less electron-rich metal fragments could induce an overall π-donor character in dipyridocarbenes and could thus proof the “built-in umpolung” [39] ability proposed by Weiss and co-workers. This property might be advantageous to stabilize low coordinated metal fragments in higher oxidation states, for example intermediates of catalytic reactions.

## Experimental

**General information.** All reactions were carried out under an inert argon atmosphere in dried and degassed solvents using standard Schlenk techniques. All metal complexes were handled in an MBraun glovebox with a nitrogen atmosphere. Solvents were dried according to standard procedures. [Rh(dipiy)Cl(cod)] (**1a**), [Rh(dipiy^tBu^)Cl(cod)] (**1b**) [56] and the dipyridoimidazolium salt dipiy^tBu^*HBF_4_ [41] were prepared according to literature procedures. NMR spectra were recorded using Bruker instruments (DRX-250, 300 or 500). ^1^H and ^13^C NMR spectra were referenced to TMS on the basis of the (residual) signal of the deuterated solvent. ^77^Se NMR shifts were calibrated towards Ph_2_Se_2_ (463 ppm) in CDCl_3_ as an external standard [80]. Medium-wall NMR tubes with a PTFE valve from Wilmad were used for the NMR experiments under CO pressure. IR spectra were recorded on a Bruker Equinox 55 FTIR spectrometer as a KBr pellet or in solution. Mass spectra were recorded on a Jeol JMS-700 and the melting point was determined with a Büchi Melting Point B 540 apparatus. The elemental analysis was carried out at Mikroanalytisches Laboratorium der Chemischen Institute of Heidelberg University. All experiments except for the synthesis and the analysis of the selenourea **4** (Institut für Anorganische Chemie of the University of Tübingen) were carried out at the Organisch-Chemisches Institut of Heidelberg University.

**Calculations.** All calculations were performed based on density functional theory at the BP86/def2-TZVP [81–85] or B3LYP/def2-TZVP [86–88] level implemented in Turbomole [89–97]. The RI-approximation [98–103] and def2-ecp [104] for Rh were used all over (in case of compounds **3a** also the D3-correction [105]). All structures were verified to be minimum structures by calculating Hessian matrices and ensuring that they have no imaginary frequency. Graphics of the MOs were prepared using POV-Ray^TM^ [106].

**NMR experiment for the in situ generation of [Rh(^13^CO)_2_Cl(dipyrido[1,2-*c*;2',1'-*e*]imidazolin-6-ylidene)] (^13C^2a).** A medium wall NMR tube was charged with a yellow solution of 6.0 mg (15 μmol) [RhCl(COD)(dipiy)] (**1a**) in 0.4 mL CD_2_Cl_2_ and pressurized with 6 bar of ^13^CO upon which a yellow precipitate formed that dissolved immediately and the solution turned greenish yellow. The signals of non-coordinated COD were observed as the only side product. ^1^H NMR (300.13 MHz, CD_2_Cl_2_) δ 7.07–7.13 (m, 4H, 2-H, 3-H, 9-H, 10-H), 7.92–7.97 (m, 2H, 1-H, 11-H), 8.92–8.97 (m, 2H, 4-H, 8-H); Contains free COD: δ = 2.31–2.36 (m, 8H, CH_2_), 5.54–5.57 (m, 4H, CH); ^13^C{^1^H} NMR (75.5 MHz, CD_2_Cl_2_) δ 117.6 (C3, C9), 118.2 (C1, C11), 120.7 (C2, C10), 127.3 (C4, C8), 184.3 (free CO), 186.3 (d, ^1^*J*_RhC_ = 54.6 Hz, *trans*-^13^CO). The signals of C6 and C11a/11b were not detected; contains free COD (δ 28.6 (CH_2_), 184.3 (CH)).

**NMR experiment of in situ generated (^13C^2a) at variable temperature.** The experiment was repeated with a sample containing 7.0 mg (17 μmol) [RhCl(COD)(dipiy)] (**1a**) in 0.4 mL CD_2_Cl_2_ at 6 bar ^13^CO. NMR spectra were recorded at −30 °C and −50 °C. Then the pressure was released and NMR spectra were recorded at −50 °C and −30 °C. Afterward the sample was opened under nitrogen atmosphere and shaken to release free ^13^CO. NMR spectra were recorded at room temperature. Finally the solution was transferred to a regular J. Young^®^ NMR tube and any residual ^13^CO removed by three freeze-thaw cycles using liquid nitrogen. The NMR spectra of the sample were then recorded at room temperature. All spectra ^13^C{^1^H} NMR (125.8 MHz, CD_2_Cl_2_, only carbonyl region) 243 K, 6 bar ^13^CO: δ 183.0 (broad peak, free and *cis*-^13^CO), 185.9 (d, ^1^*J*_RhC_ = 53.6 Hz, *trans*-^13^CO); 223 K, 6 bar ^13^CO: δ 182.8 (broad peak, free and *cis*-^13^CO), 185.7 (d, ^1^*J*_RhC_ = 53.6 Hz, *trans*-^13^CO); 223 K, 1 bar ^13^CO: δ 182.8 (free and *cis*-^13^CO), 185.7 (d, ^1^*J*_RhC_ = 53.6 Hz, *trans*-^13^CO); 243 K, 1 bar ^13^CO: δ 182.9 (free and *cis*-^13^CO), 185.9 (d, ^1^*J*_RhC_ = 53.6 Hz, *trans*-^13^CO); 298 K, 1 bar N_2_: δ 183.5 (free and *cis*-^13^CO), 186.3 (d, ^1^*J*_RhC_ = 55.5 Hz, *trans*-^13^CO); 298 K, 1 bar N_2_, after freeze-thaw cycles: δ 183.2 (dd, ^1^*J*_RhC_ = 72.8 Hz, ^2^*J*_CC_ = 6.1 Hz, *cis*-^13^CO), 186.3 (dd, ^1^*J*_RhC_ = 54.5 Hz, ^2^*J*_CC_ = 6.1 Hz, *trans*-^13^CO).

**NMR experiment for the in situ generation of [Rh(^13^CO)_2_Cl(2,10-di-*tert*-butyldipyrido[1,2-*c*;2',1'-*e*]imidazolin-6-ylidene)] (^13C^2b).** A medium-wall NMR tube was charged with a yellow solution of 10.0 mg (20.0 μmol) [RhCl(COD)(dipiy^tBu^)] (**1b**) in 0.4 mL CD_2_Cl_2_ and pressurized with 6 bar of ^13^CO. The signals of non-coordinated COD were observed as the only side product. ^1^H NMR (300.13 MHz, CD_2_Cl_2_) δ 1.39 (s, 18H, C(CH_3_)_3_), 7.13 (dd, *^3^J*_HH_ = 7.5 Hz, *^4^J*_HH_ = 1.9 Hz, 2H, 3-H, 9-H,), 7.76 (bs, 2H, 1-H, 11-H), 8.82 (d, *^3^J*_HH_ = 7.5 Hz, 2H, 4-H, 8-H). Contains free COD (δ 2.35 (br m, 8H, CH_2_), 5.55 (br m, 4H, CH); ^13^C{^1^H} NMR (75.5 MHz, CD_2_Cl_2_) δ 30.6 (C(*C*H_3_)_3_), 35.4 (*C*(CH_3_)_3_), 112.0 (C1, C11), 117.2 (C3, C9), 123.9 (C11a, C11b), 126.7 (C4, C8), 143.9 (C2, C10), 184.1 (broad peak, free and *cis*-^13^CO) 186.6 (d, ^1^*J*_RhC_ = 55.4 Hz, *trans*-^13^CO). The carbene signal C6 was not detected; contains free COD (δ 28.6 (CH_2_), 129.2 (CH)).

**Synthesis of [Rh(CO)_2_Cl(dipyrido[1,2-*c*;2’,1’-*e*]imidazolin-6-ylidene)] (2a).** In a 25 mL-size glass autoclave was dissolved [RhCl(COD)(dipiy)] (**1a**) (60.0 mg, 150 μmol) in 5 mL dichloromethane and pressurized with CO (8 bar) upon which an immediate color change to green was observed. Afterwards the pressure was released and all volatiles removed in vacuo. The light yellow residue was washed two times with pentane (1 mL each) and dried in vacuo to obtain the carbonyl complex **2a** in 93% yield (47.0 mg, 130 μmol). Mp 259–262 °C (dec); ^1^H NMR (300.13 MHz, CD_2_Cl_2_) δ 7.07–7.14 (m, 4H, 2-H, 3-H, 9-H, 10-H), 7.93–7.96 (m, 2H, 1-H, 11-H), 8.93–8.96 (m, 2H, 4-H, 8-H); ^13^C{^1^H} NMR (−30 °C, 75.5 MHz, CD_2_Cl_2_) δ 117.3 (C3, C9), 117.8 (C1, C11), 120.2 (C2, C10), 123.4 (C11a, C11b), 126.4 (C4, C8), 152.2 (d, *^1^J*_RhC_ = 43.6 Hz, C6), 182.5 (d, C_CO_, *^1^J*_RhC_ = 72.7 Hz, *cis*-CO), 185.9 (d, *^1^J*_RhC_ = 54.0 Hz, *trans*-CO); IR (KBr, cm^−1^) 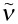: 3105 (w), 3058 (w), 2963 (w), 2073 (s, CO), 1993 (s, CO), 1622 (w), 1355 (w), 1331 (w), 739 (m), 704 (w); (CH_2_Cl_2_, cm^−1^) 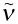: 2082 (m, CO), 2003 (m, CO); (DMSO, cm^−1^) 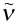: 2064 (m, CO), 1984 (m, CO); MS (FD^+^, LIFDI^+^ in CH_2_Cl_2_) *m*/*z*: 362.0 [M^+^]; anal. calcd for C_13_H_8_ClN_2_O_2_Rh: C, 43.06; H, 2.39; N, 7.73; found: C, 42.88; H, 2.22; N, 7.65.

**Synthesis of [Rh(CO)_2_Cl(2,10-di-*tert*-butyldipyrido[1,2-*c*;2’,1’-*e*]imidazolin-6-ylidene)] (2b).** [RhCl(COD)(dipiy^tBu^)] (**1b**) (15.0 mg, 30.0 μmol) was dissolved in 2.5 mL dichloromethane and pressurized with CO (8 bar) in a 10 mL-size glass autoclave. After 10 min the pressure was released and all volatiles were removed in vacuo. The residue was washed with pentane (1 mL) and dried in vacuo to obtain about 50% (7.0 mg, 15 μmol) of the carbonyl complex **2b** as a yellow solid. ^13^C{^1^H} NMR (243 K, 125.8 MHz, CD_2_Cl_2_) δ 30.0 (C(*C*H_3_)_3_), 35.0 (*C*(CH_3_)_3_), 111.5 (C1, C11), 116.9 (C3, C9), 123.2 (C11a, C11b), 126.1 (C4, C8), 143.2 (C2, C10), 150.3 (d, *^1^J*_RhC_ = 44.4 Hz, C6), 182.8 (d, *^1^J*_RhC_ = 75.8 Hz, *cis*-CO), 186.1 (d, *^1^J*_RhC_ = 53.6 Hz, *trans*-CO); IR (KBr, cm^−1^) 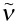: 2962 (s), 2868 (m), 2075 (s, CO), 1997 (s, CO), 1659 (w), 1533 (w), 1475 (w), 1366 (w), 1335 (w), 1300 (w), 1267 (m), 964 (m), 873 (w), 789 (m), 638 (m), 590 (m); HRMS (FAB^+^ in NBA) *m*/*z*: 446.0612 [M(^35^Cl) − CO^+^] (calcd 446.0632), 448.0596 [M(^37^Cl) − CO^+^] (calcd 448.0603), 474.0584 [M(^35^Cl)^+^] (calcd 474.0581), 476.0581 [M(^37^Cl)^+^] (calcd 476.0552).

**Synthesis of 2,10-di-*tert*-butyldipyrido[1,2-*c*:2′,1′-*e*]imidazolin-6-selenone (4b)**. A suspension of 2,10-di-*tert*-butyldipyrido[1,2-*c*:2′,1′-*e*]imidazolium tetrafluoroborate [41] (40.5 mg, 110 μmol) and selenium (32.1 mg, 407 μmol) in 3 mL of tetrahydrofuran was cooled to −35 °C and a solution of potassium *tert*-butoxide (14.9 mg, 133 μmol) in 1 mL tetraydrofuran was added. After 30 min the deep red suspension was warmed up to room temperature and stirred overnight. The solvent was removed in vacuo and the residue suspended in 7 mL of dichloromethane. After filtration through a pipette containing glass wool and 3 cm of Celite^®^, the red solution was concentrated to dryness in vacuo to yield 33.2 mg (84%) of the product as a red solid. ^1^H NMR (250.13 MHz, CDCl_3_) δ 1.38 (s, 18H, *t*-Bu), 7.19 (dd, ^3^*J*_HH_ = 7.7 Hz, ^4^*J*_HH_ = 1.9 Hz, 2H, 3-H, 9-H), 7.65 (dd, ^4^*J*_HH_ = 1.9 Hz, ^5^*J*_HH_ = 1.0 Hz, 2H, 1-H, 11-H), 8.73 (dd, ^3^*J*_HH_ = 7.7 Hz, ^5^*J*_HH_ = 1.0 Hz, 2H, 4-H, 8-H); ^13^C{^1^H} NMR (62.9 MHz, CDCl_3_) δ 30.2 (C(*C*H_3_)_3_), 34.8 (*C*(CH_3_)_3_), 111.0 (C1, C11), 116.2 (C3, C9), 120.6 (C11a, C11b), 124.2 (C4, C8), 131.9 (C6), 142.6 (C2, C10); ^77^Se NMR (47.70 MHz, CDCl_3_) δ −55.8 (s, Se); HRMS (ESI^+^) *m*/*z*: 360.11023 [M^+^] (calcd 360.10992).

## Supporting Information

File 1 NMR spectra of compounds **2a**, **2b** and **4b** as well as details of the DFT calculations.
