# Supplementary material for: Experimental and theoretical investigations on the high-electron donor character of pyrido-annelated N-heterocyclic carbenes
Source: Beilstein J Org Chem. 2016 Aug 23;12:1884–96. doi: 10.3762/bjoc.12.178 (PMC5082490; doi:10.3762/bjoc.12.178)
Supplement: File 1 — NMR spectra of compounds 2a, 2b and 4b as well as details of the DFT calculations. [file Beilstein_J_Org_Chem-12-1884-s001.pdf]

Supporting Information

for

Experimental and theoretical investigations on the high-electron donor character of pyrido-annelated N-heterocyclic carbenes

Michael Nonnenmacher[1], Dominik M. Buck[2] and Doris Kunz*[1,2]

Address: [1]Organisch-Chemisches Institut, Ruprecht-Karls Universität Heidelberg, Im Neuenheimer Feld 250, D-69120 Heidelberg, Germany and [2]Institut für Anorganische Chemie, Eberhard Karls Universität Tübingen, Auf der Morgenstelle 18, D-72076 Tübingen, Germany (current address of corresponding author)

Email: Doris Kunz – Doris.Kunz@uni-tuebingen.de

* Corresponding author

NMR spectra of compounds 2a, 2b and 4b as well as details of the DFT calculations

Content

1. NMR Spectra S2

2. DFT Calculations S5

2.1. xyz-Coordinates of the reaction intermediates 3a S5

2.2. Thermochemical data for the ^{13}CO exchange reaction S7

2.3. Highest occupied molecular orbitals and the two MOs below (number of MO) of the carbenes I – III and dipiy. S8

2.4. Selected MOs of carbenes I-III and dipiy and their Rh complexes I-Rh – III-Rh and 2a S8

2.5. xyz-Coordinates of carbenes I-III and dipiy and their Rh-complexes S15

1. NMR Spectra

Compound 2a

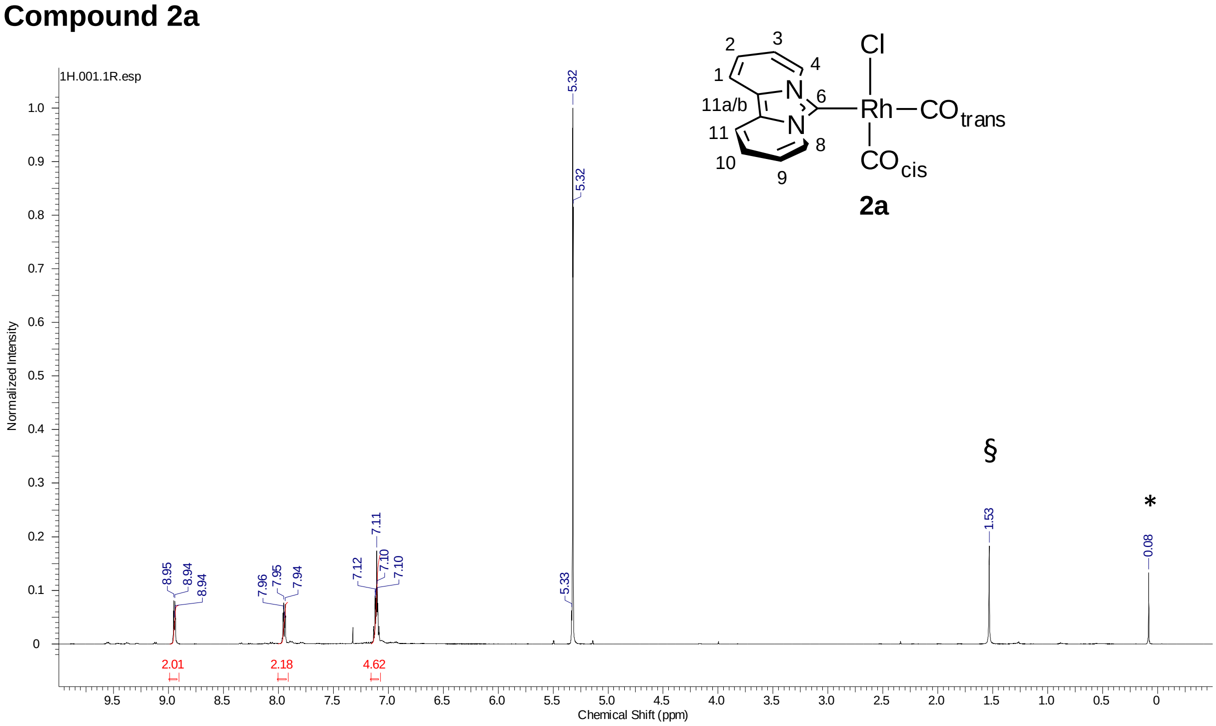

Figure S1: ^{1}H NMR spectrum (500.13 MHz, $CDCl_3$) of the isolated complex **2a** (§ residual water from CD_2Cl_2; * silicon grease).

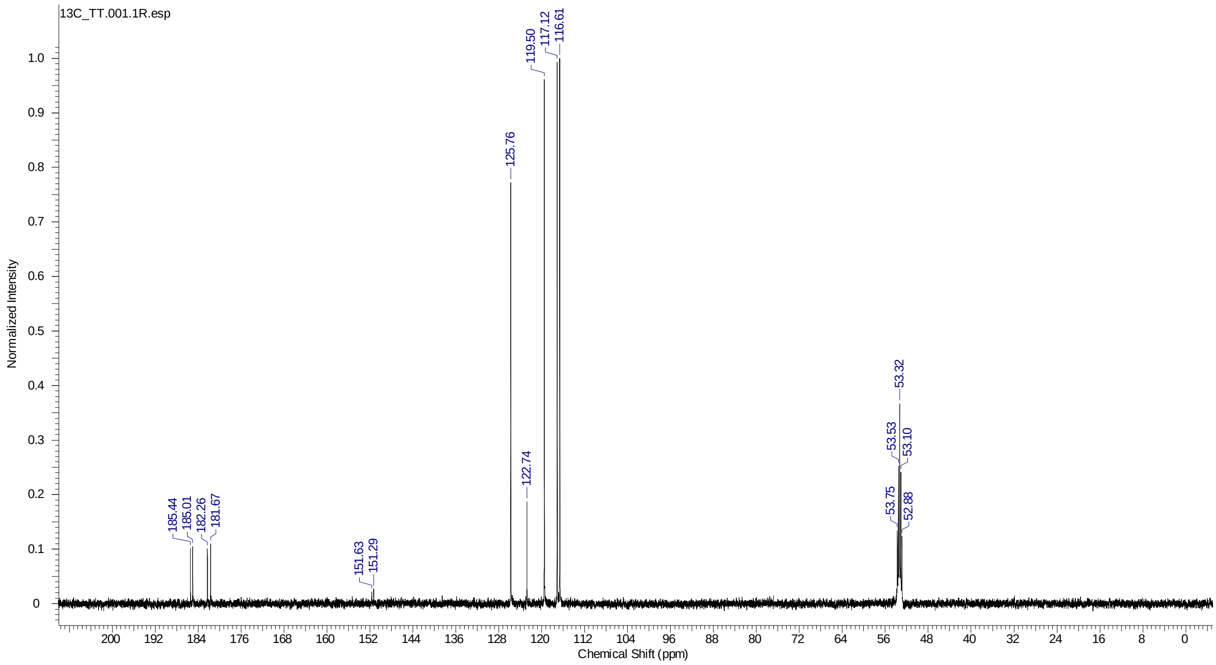

Figure S2: ^{13}C NMR spectrum (125.76 MHz, -30 °C, CD_2Cl_2) of the isolated complex **2a**.

Compound 2b

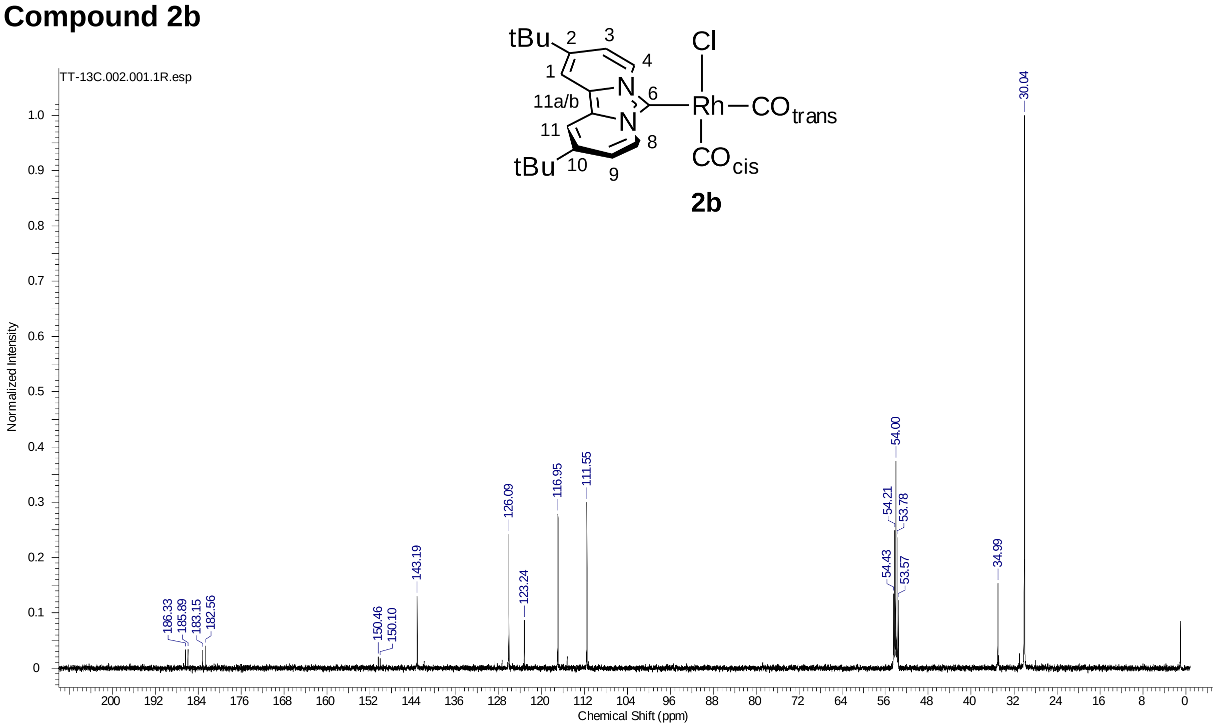

Figure S3: ^{13}C NMR spectrum (125.76 MHz, -30 °C, CD_2Cl_2) of the isolated complex **2b**.

Compound 4b

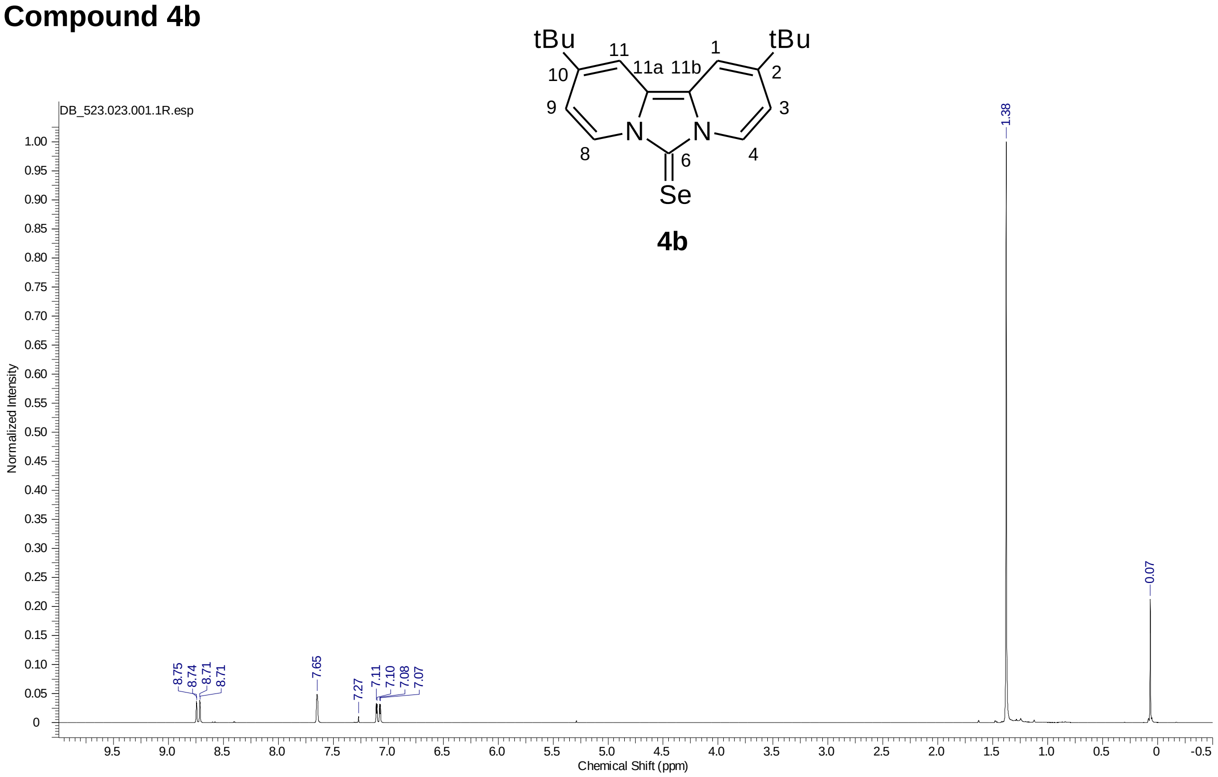

Figure S4: ^{1}H NMR spectrum (250.13 MHz, $CDCl_3$) of the selenourea **4b**.

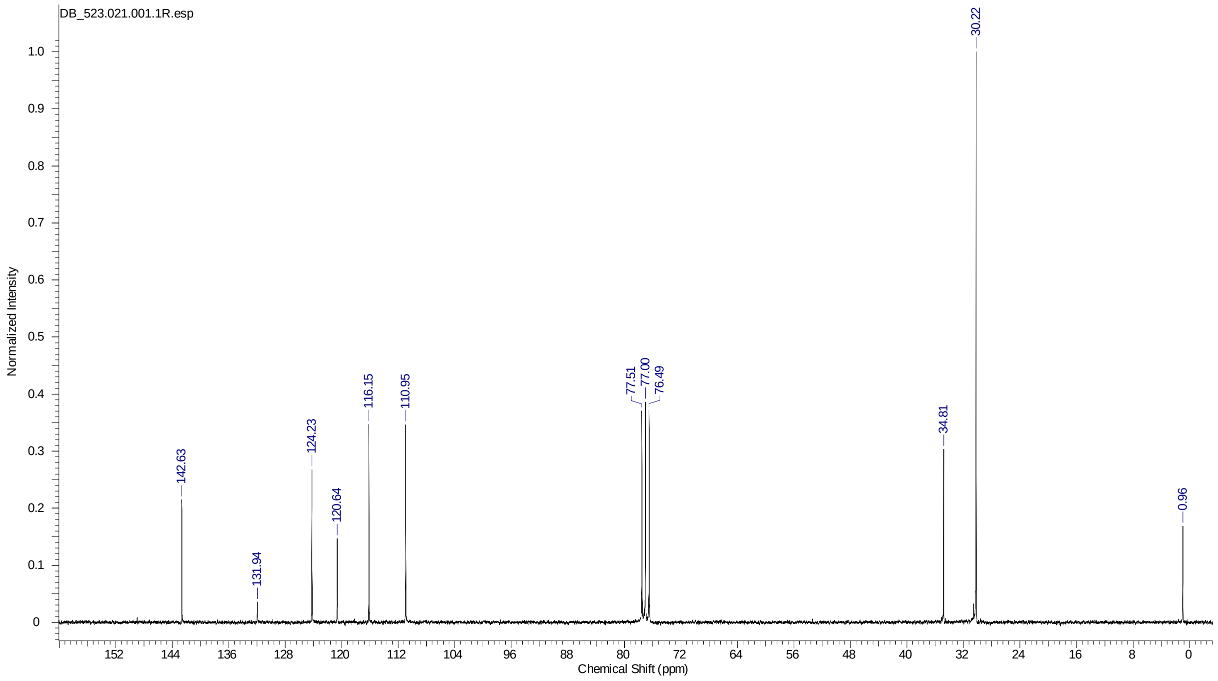

Figure S5: ^{13}C NMR spectrum (62.90 MHz, $CDCl_3$) of the selenourea **4b**.

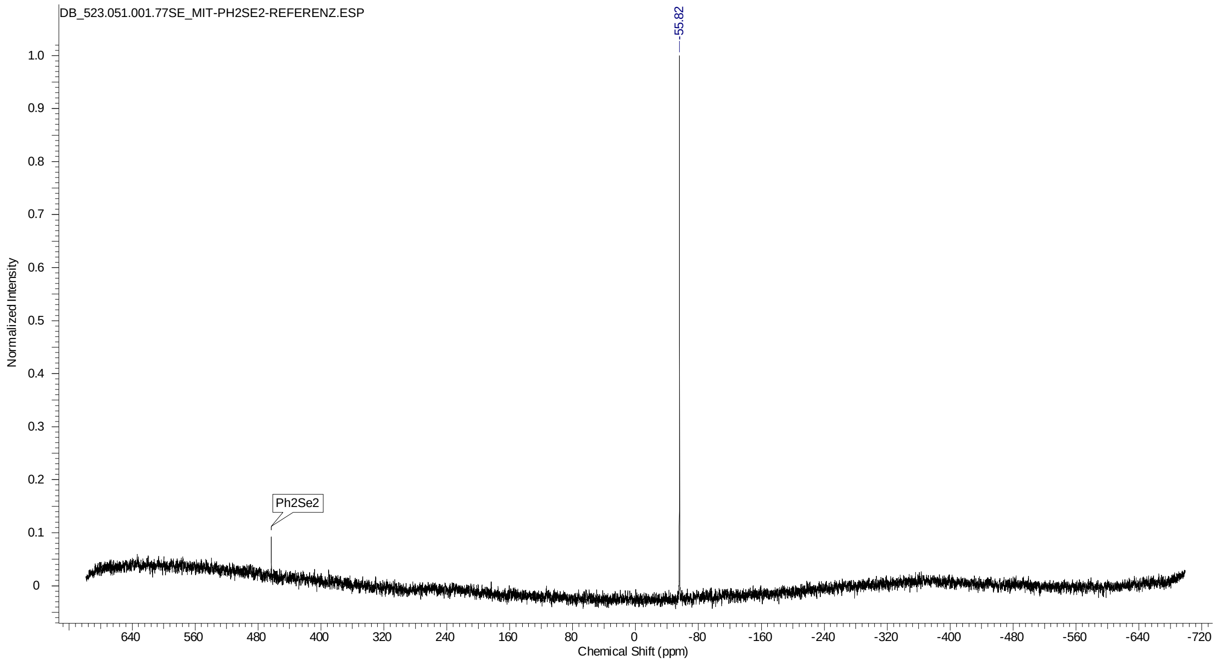

Figure S6: ^{77}Se NMR spectrum (47.70 MHz, $CDCl_3$) of the selenourea **4b**.

2. DFT Calculations

2.1. xyz-Coordinates of the reaction intermediates 3a

3a NHC/CO apic

BP86/def2-TZVP + Disp

SCF-energy : -1444.9915835 H
SCF + E(vib0) : -1444.8054643 H

C -0.041451 -0.438731 0.109425
N 0.336643 0.923871 0.093051
C -0.738823 1.770399 0.208341
N -1.816911 0.920020 0.284770
C -1.434288 -0.439485 0.231205
C 0.936596 -1.443583 0.009377
C 2.261940 -1.092186 -0.112742
C 2.618115 0.287272 -0.141335
C 1.674421 1.268091 -0.041531
H 0.619605 -2.485564 0.030286
H 3.033506 -1.856197 -0.190619
H 3.659934 0.585324 -0.246741
H 1.875997 2.335775 -0.065256
C -3.736471 -1.097002 0.407793
C -2.410959 -1.449666 0.296446
C -3.153822 1.269420 0.394103
C -4.094451 0.280332 0.455159
H -4.508640 -1.863359 0.458855
H -2.093519 -2.490745 0.253804
H -3.354122 2.346363 0.445012
H -5.138415 0.576648 0.543644
Rh -0.619649 3.855943 0.201346
O -0.489728 6.912541 0.149311
C -0.522462 5.766349 0.168126
O -0.050170 3.480195 -2.777273
Cl -3.058049 4.506488 0.810467
C 0.352624 3.702456 1.845037
O 1.021014 3.545917 2.774445
C -0.327528 3.665969 -1.669662

3a Cl/CO apic

BP86/def2-TZVP + Disp

SCF-energy : -1444.9844700 H
SCF + E(vib0) : -1444.7990447 H

C -0.016136 -0.496557 0.104254
N 0.413904 0.849471 0.056585
C -0.625154 1.745883 0.118532
N -1.730931 0.939367 0.203573
C -1.410017 -0.441005 0.195691
C 0.927240 -1.538916 0.049164
C 2.265418 -1.231844 -0.046476
C 2.673416 0.133277 -0.087066
C 1.762600 1.148656 -0.032391
H 0.575824 -2.569491 0.084421
H 3.012056 -2.023453 -0.086715
H 3.728919 0.391161 -0.153534
H 2.004541 2.206817 -0.028424
C -3.745244 -0.990094 0.364275
C -2.434506 -1.401121 0.272718
C -3.052456 1.341122 0.310904
C -4.042019 0.404648 0.388179
H -4.551599 -1.718980 0.424542
H -2.165606 -2.456913 0.260470
H -3.209747 2.415590 0.342223
H -5.071789 0.747322 0.474992
Rh -0.494031 3.883082 0.199384
C 1.015934 4.812523 -0.585439
C -1.527049 4.024623 -1.330791
O -2.536296 5.196222 2.141982
Cl 0.908949 3.553357 2.144605
O -2.173955 4.086688 -2.286715
O 1.926559 5.376629 -1.016325
C -1.799645 4.691067 1.412953

3a (III) III/CO apic

BP86/def2-TZVP

SCF-energy : -1216.2269241
SCF + E(vib0) : -1216.0765779

C -0.072062 -0.511912 -0.037153
N 0.421579 0.783272 -0.065394
C -0.583464 1.692186 0.114916
N -1.714081 0.936800 0.227002
C -1.416165 -0.412195 0.143657
C 1.833251 1.090366 -0.267475
H 2.292078 0.269702 -0.830822
C -3.080884 1.451495 0.332677
H -3.453981 1.729625 -0.662060
Rh -0.506017 3.774347 0.186601
Cl -1.874129 3.828511 2.351985
C -0.738183 5.666414 0.336832
C -1.159271 3.736600 -1.580443
O -0.925865 6.792074 0.452099
O -1.463032 3.719062 -2.699959
H 2.352462 1.205001 0.692857
H 1.922277 2.018079 -0.841230
H -3.713273 0.660808 0.751817
H -3.079948 2.324649 1.000314
H 0.569763 -1.377427 -0.142528
H -2.180508 -1.175295 0.218577
C 1.266460 3.877240 0.987097
O 2.334142 4.070156 1.380657

3a (III) Cl/CO apic

BP86/def2-TZVP

SCF-energy : -1216.2204659
SCF + E(vib0) : -1216.0709012

C -0.077575 -0.522565 -0.020822
N 0.376044 0.785974 -0.054024
C -0.658687 1.672089 0.022562
N -1.770738 0.880433 0.093685
C -1.434228 -0.465223 0.069254
C 1.786333 1.159590 -0.133569
H 2.391200 0.249158 -0.058444
C -3.142520 1.362799 0.186882
H -3.632787 1.337384 -0.796118
Rh -0.505065 3.826253 0.138136
Cl 0.667754 3.464270 2.226048
C -1.800614 4.809575 1.202868
C -1.415619 4.021323 -1.461632
O -2.519544 5.438877 1.850186
O -1.983835 4.131493 -2.462709
H 2.024667 1.839291 0.694007
H 1.992046 1.656722 -1.089535
H -3.703123 0.733987 0.889238
H -3.120159 2.392490 0.557289
H 0.594543 -1.371169 -0.052295
H -2.175436 -1.252645 0.126829
C 1.138265 4.638759 -0.526922
O 2.101844 5.170746 -0.872798

2.2. Thermochemical data for the ^{13}CO exchange reaction

Table S1: Thermochemical data for the reaction of **2a** and CO to complex **3a** at 1 bar and 6 bar at room temperature as well as at 6 bar at -50 °C.

	Product	Educt 1	Educt 2
	**3a NHC/CO trans**	**CO**	**2a**
SCF	-1444,9505728	-113,3653363	-1331,5791984
SCF+E_{vib}0	-1444,7645456	-113,3604949	-1331,4002511
H (298K, 1 bar)	0,2071010	0,0081470	0,1972570
SCF+H (298K, 1 bar)	-1444,7434718	-113,3571893	-1331,3819414
G(298K, 1bar)	0,1338510	-0,0143280	0,1309590
SCF+G (298K, 1 bar)	-1444,8167218	-113,3796643	-1331,4482394
$\Delta H_{R\ (298\ K,\ 1\ bar)}$	-0,0043411	-11,3975564	kJ/mol
**$\Delta G_{R\ (298\ K,\ 1\ bar)}$**	0,0111819	**29,3580742**	**kJ/mol**
H(298K, 6bar)	0,2071010	0,0081470	0,1972570
SCF+H (298K, 6 bar)	-1444,7434718	-113,3571893	-1331,3819414
G(298K, 6 bar)	0,1355430	-0,0126360	0,1326500
SCF+G (298K ,6 bar)	-1444,8150298	-113,3779723	-1331,4465484
$\Delta H_{R\ (298\ K,\ 6\ bar)}$	-0,0043411	-11,3975564	kJ/mol
**$\Delta G_{R\ (298\ K,\ 6\ bar)}$**	0,0094909	**24,9183543**	**kJ/mol**
H(223K, 6bar)	0,1989970	0,0073150	0,1901460
SCF+H (223K, 6 bar)	-1444,7515758	-113,3580213	-1331,3890524
G(223K, 6 bar)	0,1523900	-0,0075230	0,1478880
SCF+G (223K ,6 bar)	-1444,7981828	-113,3728593	-1331,4313104
$\Delta H_{R\ (223\ K,\ 6\ bar)}$	-0,0045021	-11,8202618	kJ/mol
**$\Delta G_{R\ (223\ K,\ 6\ bar)}$**	0,0059869	**15,7186037**	**kJ/mol**

2.3. Highest occupied molecular orbitals and the two MOs below (number of MO) of the carbenes I – III and dipiy.

The energetic stabilization of the σ-orbital due to a more acute N-C-N angle and the increase of the energy of the highest occupied π-MO lead to a switch in the order for the dipyrido carbene dipiy.

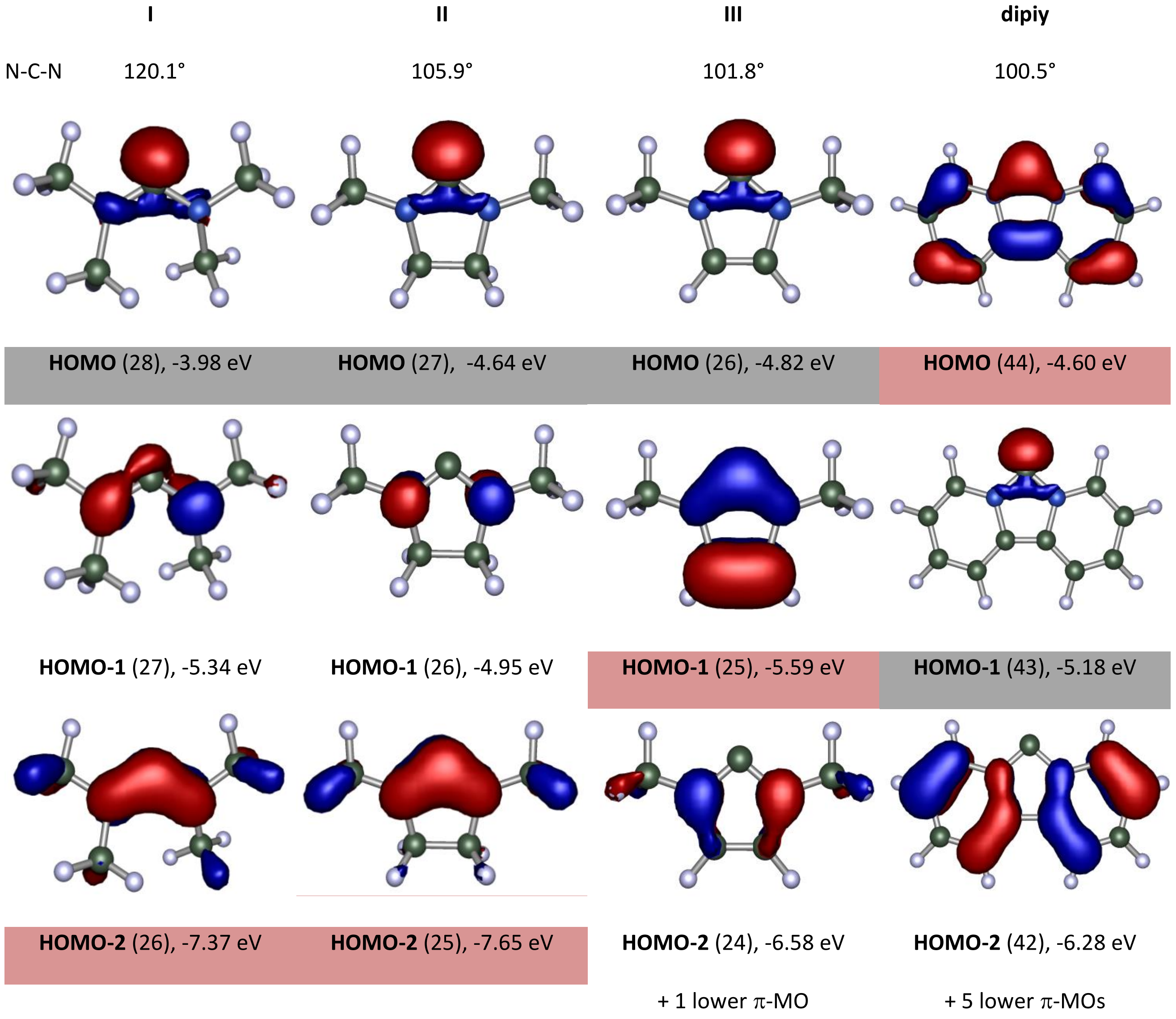

2.4. Selected MOs of carbenes I-III and dipiy and their Rh complexes I-Rh – III-Rh and 2a

For the Rh-complexes **I-Rh** – **III-Rh**, the highest occupied molecular orbital is the d_{z^2}-orbital, while for **2a** it is the non-bonding π-orbital of the **dipiy** ligand (it is strongly mixed with the d_{z^2} due to a small energy difference when using the BP86 functional, but separated using B3LYP; all other orbitals are identical in order and shape for both functionals).

**I** HOMO-LUMO = 3.934 eV	**I-Rh**
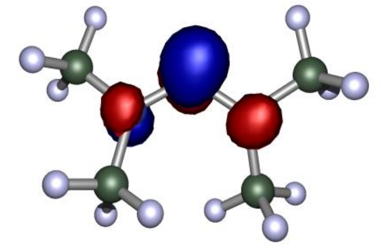 LUMO (29); -0.00182 H; -0.0495 eV	
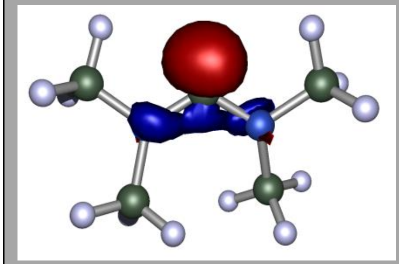 HOMO (28); -0.14639 H; -3.9836 eV	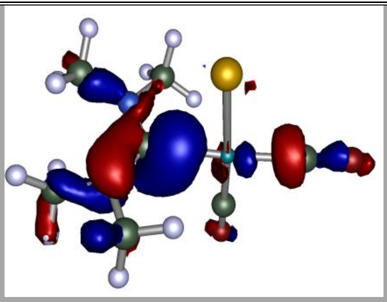 HOMO-8 (51); -0.2984 H; -8.1200 eV
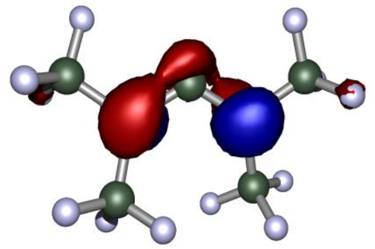 HOMO-1 (27); -0.19619 H; -5.3387 eV	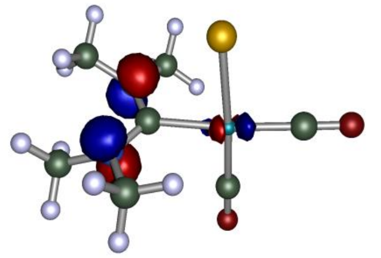 HOMO-3 (56); -0.2235 H; -6.0818 eV
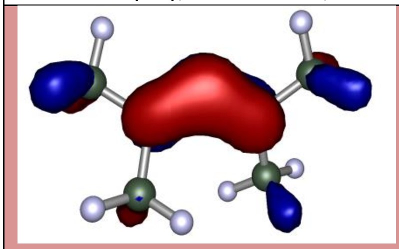 HOMO-2 (26); -0.27066 H; -7.3652 eV	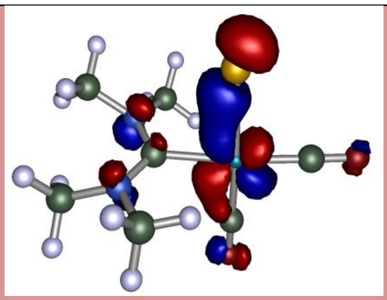 HOMO-6 (53); -0.2659 H; -7.2356 eV
	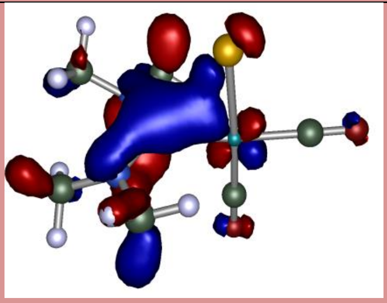 HOMO-9 (50); -0.3223 H; -8.7703 eV

**II** HOMO-LUMO = 4.58 eV	**II-Rh**
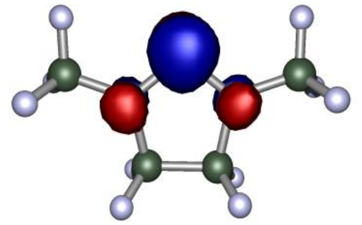 LUMO(28) 0.05966 H; -0.0645 eV	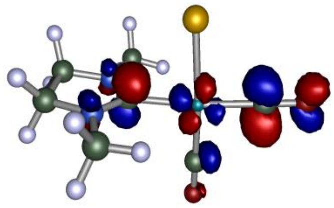 LUMO+5(64) -0.0271 H;
	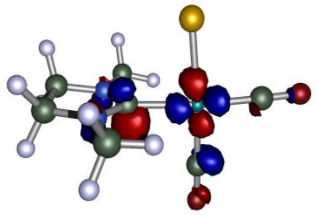 LUMO+3(62) -0.0388 H
	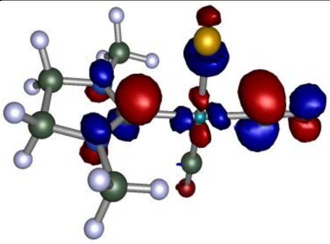 LUMO+1(60) -0.0664 H;
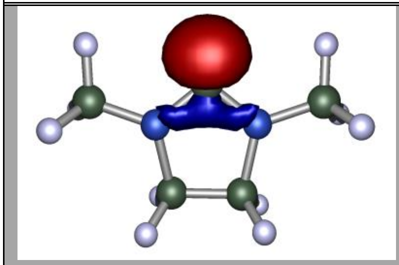 HOMO(27) -0.17069 H; -4.6448 eV	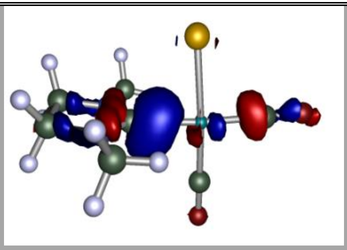 HOMO-8 (50) -0.3019 H; -8.2151 eV
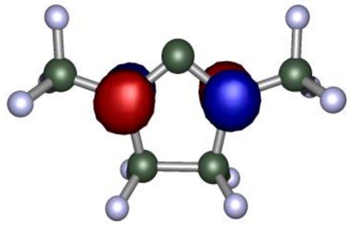 HOMO-1(26) -0.18206 H; -4.9542 eV	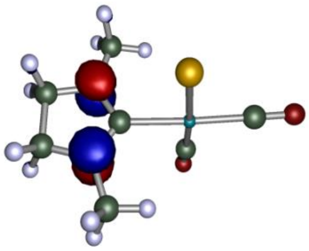 HOMO-3(55) -0.22021 H; -5.9922 eV
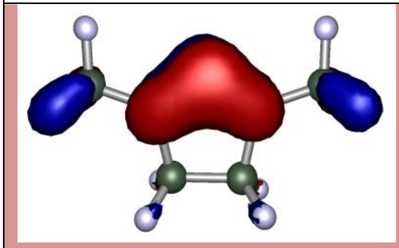 HOMO-2(25) -0.28096 H; -7.6453 eV	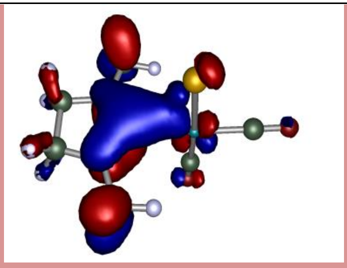 HOMO-9 (49) -0.3335 H; -9.0750 eV

<table>
<tr><th>III HOMO-LUMO = 4.468</th><th>III-Rh</th></tr>
<tr><td rowspan="3">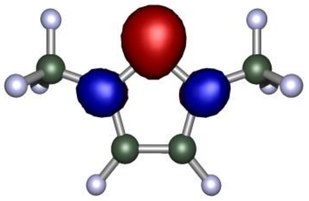
LUMO+1 (28) 0.00182 H; 0.0496 eV</td><td>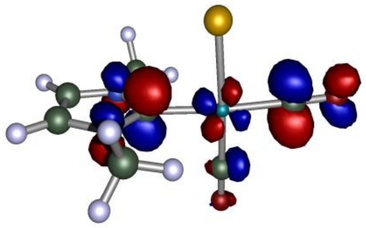
LUMO+6 (64) -0.0263 H; -0.7157 eV</td></tr>
<tr><td>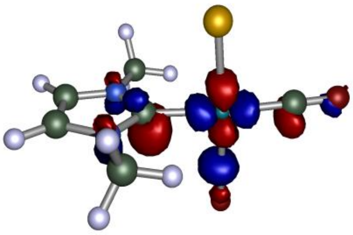
LUMO+4 (62) -0.0370 H; -1.0068 eV</td></tr>
<tr><td>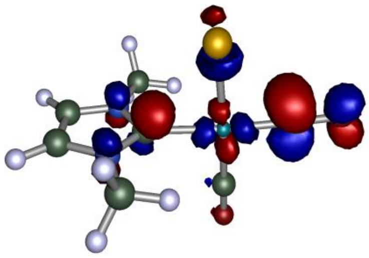
LUMO+1 (59) -0.0652 H; -1.7742 eV</td></tr>
<tr><td>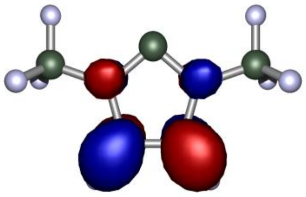
LUMO (27) -0.01291 H; -0.3514 eV</td><td>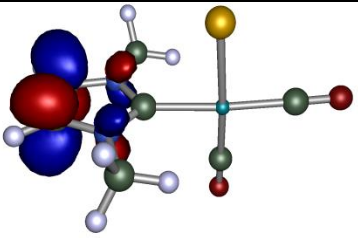
LUMO+2 (60) -0.0445 H; -1.2109 eV</td></tr>
<tr><td>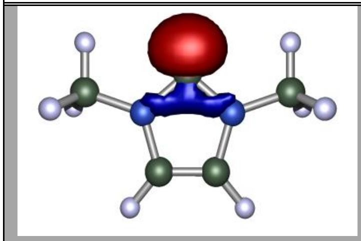
HOMO (26) -0.17711 H; -4.8195 eV</td><td>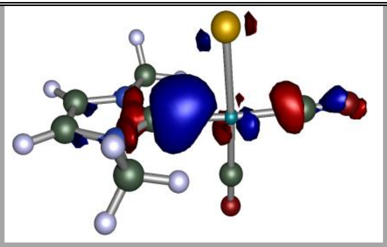
HOMO-9 (48) -0.3116; -8.4791 eV</td></tr>
<tr><td>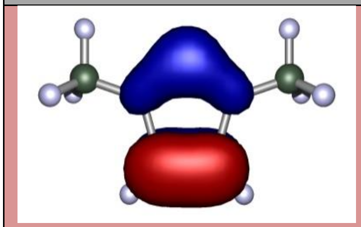
HOMO-1 (25) -0.20537 H; -5.5885 eV</td><td>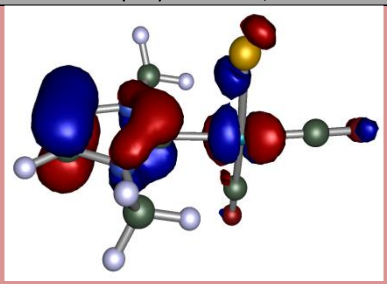
HOMO-4 (53) -0.2360 H; -6.4219 eV</td></tr>
</table>

	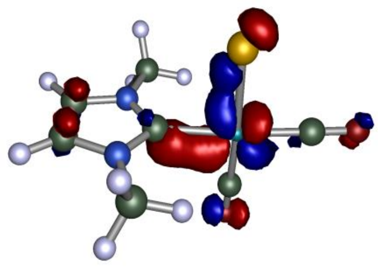<br>HOMO-8 (49) -0.2884 H; -7.8478 eV
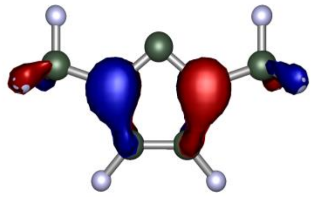<br>HOMO-2 (24) -0.24180 H; -6.5797 eV	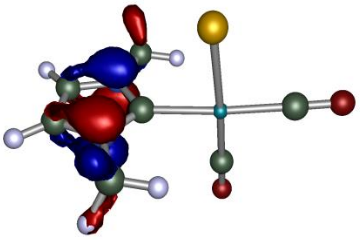<br>HOMO-7 (50) -0.2808 H; -7.6410 eV
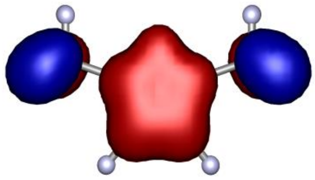<br>HOMO-3 (23) -0.33721 H; -9.1759 eV	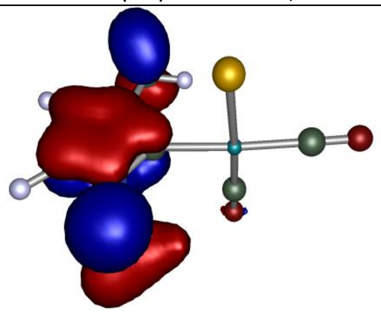<br>HOMO-10 (47) -0.3708 H; -10.0901 eV

**dipiy**	**2a**
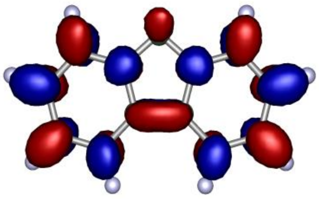<br>LUMO+7 (52) +0.0561	
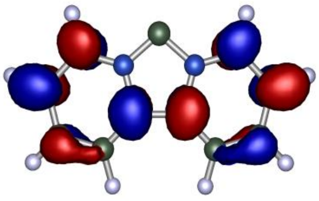<br>LUMO+3 (48) -0.02019 H; -0.5493 eV	

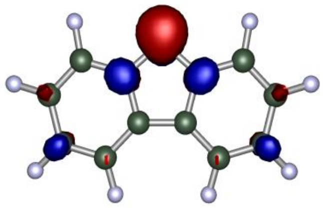<br>LUMO+2 (47) -0.02144 H;- 0.5835 eV	
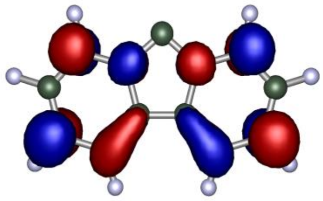<br>LUMO+1 (46) -0.07920 H; -2.1552 eV	
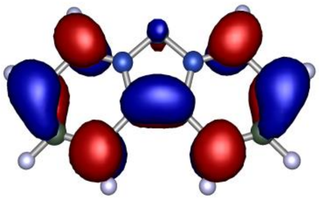<br>LUMO (45) -0.08183 H; -2.2269 eV	
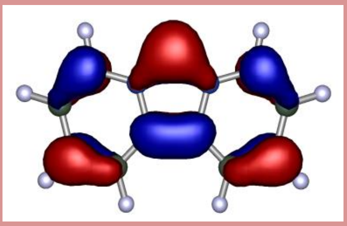<br>HOMO (44) -0.16895 H; -4.5975 eV	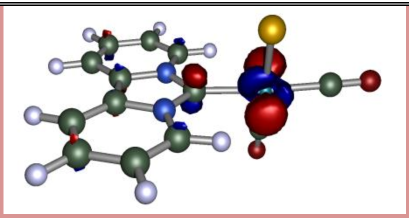<br>HOMO (75) -0.1931 H; -5.2545 eV
	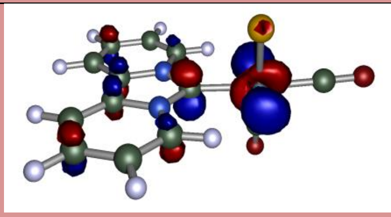<br>HOMO-1 (74) -0.1940 H; -5.2791 eV
	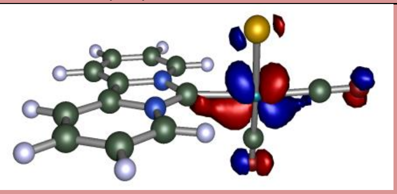<br>HOMO-8 (67) -0.2796 H; -7.6084 eV
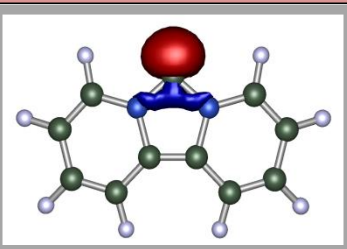<br>HOMO-1 (43)-0.19041 H; -5.1813 eV	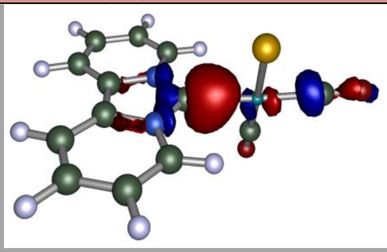<br>HOMO-11 (64) -0.3117 H; -8.4819 eV

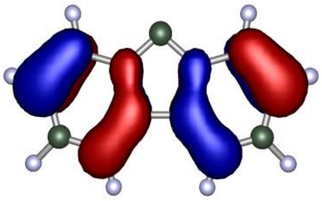<br>HOMO-2 (42) -0.23089 H; -6.2829 eV	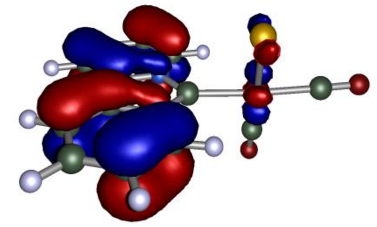<br>HOMO-5 (70) -0.2543 H; -6.9199 eV
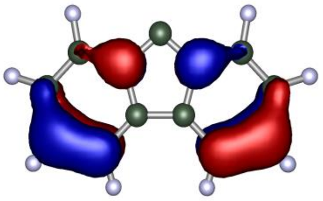<br>HOMO-3 (41) -0.27805 H; -7.5661 eV	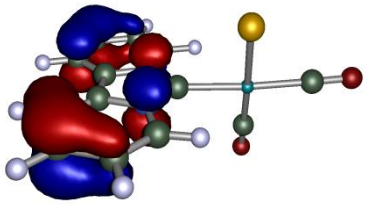<br>HOMO-9 (66) -0.3032 H; -8.2506 eV
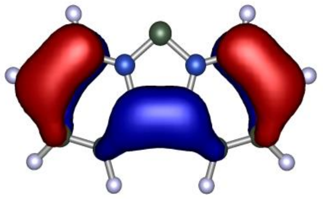<br>HOMO-4 (40) -0.28406 H; -7.7298 eV	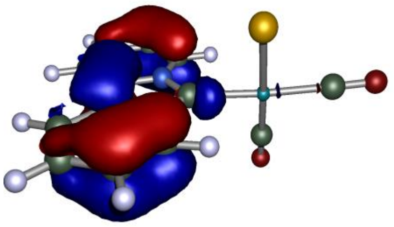<br>HOMO-10 (65) -0.3069 H; -8.3512 eV
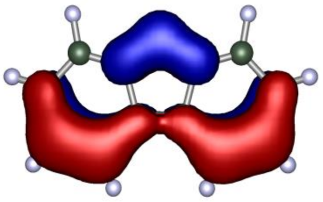<br>HOMO-5 (39) -03062 H; -8.3321 eV	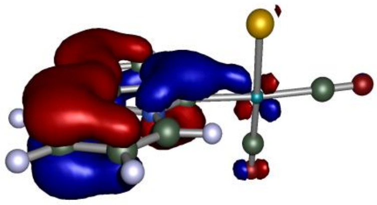<br>HOMO-12 (63) -0.3356 H; -9.1322 eV
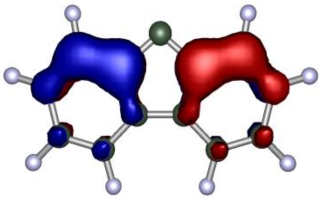<br>HOMO-10 (34) -0.3658 H; -9.9539 eV	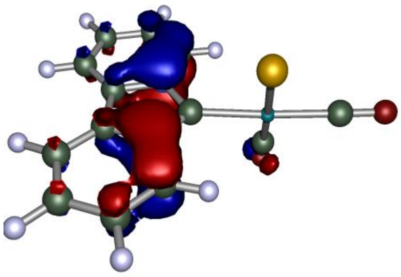<br>HOMO-17 (58) -0.3936 H; -10.71048 eV
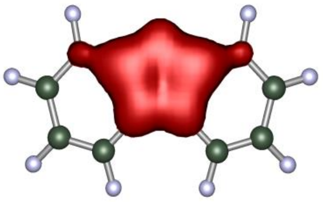<br>HOMO-14 (30) -0.4291 H; -11.676 eV	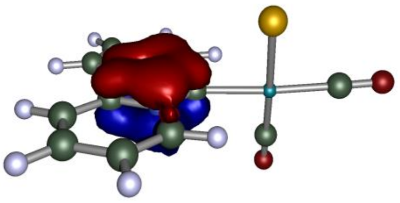<br>HOMO-29 (46) -0.4639 H; -12.6235 eV

2.5. xyz-Coordinates of carbenes I-III and dipiy and their Rh-complexes

Ia

BP86/def2-TZVP

SCF-energy : -621.9187423
SCF + E(vib0) : -621.5353194

C 0.409317 -0.858082 -0.142512
N 0.255570 0.627791 -0.020959
C -0.893868 1.342497 0.014498
N -2.129578 0.835425 0.180912
C -2.625349 -0.506142 0.640737
C 1.459224 1.523106 -0.104225
C 1.927318 1.804096 -1.539813
C -3.213002 1.859888 -0.004836
C -3.121983 2.978476 1.036210
C 2.611925 1.125114 0.823274
H 1.051734 2.468586 0.273284
C -3.207720 2.416191 -1.430921
H -4.164538 1.326301 0.140048
C -3.387812 -1.255725 -0.459103
C -3.448255 -0.408792 1.935677
C 0.733843 -1.553517 1.190872
C 1.373955 -1.319712 -1.244934
H -0.575760 -1.202958 -0.477117
H -1.737884 -1.086914 0.903071
H 2.263872 0.993333 1.856866
H 3.359980 1.931776 0.822287
H 3.130638 0.207867 0.511075
H -3.306239 1.609657 -2.172078
H -2.260989 2.941033 -1.615775
H -4.043302 3.118316 -1.572053
H -3.673717 -1.422422 2.299357
H -2.887437 0.117428 2.719407
H -4.408238 0.106616 1.793694
H 2.422542 -1.082087 -1.025831
H 1.300546 -2.414015 -1.335421
H 1.112212 -0.883937 -2.217289
H 1.773486 -1.378469 1.495171
H 0.084741 -1.193715 2.001045
H 0.593829 -2.641870 1.097010
H -2.149804 3.479859 0.940013
H -3.925487 3.713480 0.880485
H -3.209615 2.584597 2.058461
H 1.063065 2.062902 -2.167230
H 2.610591 2.666973 -1.535944
H 2.460971 0.963627 -2.000516
H -3.690742 -2.254287 -0.108635
H -2.768184 -1.379334 -1.358462
H -4.301258 -0.717969 -0.752861

Ia-Rh

BP86/def2-TZVP

SCF-energy : -1419.8435378
SCF + E(vib0) : -1419.4398807

C 0.159353 -0.667927 -0.728892
N 0.231434 0.725517 -0.187505
C -0.876095 1.513075 -0.083202
N -2.085932 0.931237 0.136828
C -2.246066 -0.226741 1.081283
C 1.547778 1.367240 0.118334
C 2.652488 1.162617 -0.919347
C -3.372333 1.573251 -0.282436
C -3.716844 2.906527 0.388034
C 2.017641 1.042743 1.542374
H 1.301197 2.448082 0.111177
C -3.465843 1.655849 -1.810149
H -4.134288 0.851086 0.042258
C -3.044016 -1.404326 0.502675
C -2.853558 0.245041 2.410672
C 0.926136 -1.716992 0.082670
C 0.519982 -0.732885 -2.226195
H -0.899159 -0.927349 -0.683668
H -1.227533 -0.562416 1.302471
H 1.224239 1.268121 2.266589
H 2.880074 1.679229 1.788502
H 2.332506 -0.003332 1.656075
H -3.268481 0.679769 -2.275370
H -2.750476 2.384141 -2.212060
H -4.475045 1.977678 -2.104285
H -2.823608 -0.584446 3.132574
H -2.285734 1.091970 2.817968
H -3.905205 0.543987 2.298039
H 1.596174 -0.664432 -2.415294
H 0.174293 -1.698021 -2.625089
H 0.014727 0.064295 -2.786650
H 2.013479 -1.585684 0.008959
H 0.653001 -1.699167 1.146242
H 0.690020 -2.715947 -0.311316
H -3.024510 3.706869 0.079206
H -4.736903 3.195552 0.093841
H -3.666907 2.841950 1.480946
H 2.330633 1.455391 -1.926716
H 3.495998 1.809744 -0.640126
H 3.036333 0.133497 -0.951815
H -2.984266 -2.256550 1.194475
H -2.670267 -1.736542 -0.475319
H -4.109211 -1.158275 0.388065
Rh -0.619349 3.620041 -0.071800
C -0.368307 5.494989 0.133657
Cl -0.715346 3.433225 2.309566
C -0.440386 3.676261 -1.895957
O -0.291792 3.717833 -3.047760
O -0.207138 6.625661 0.278438

I

BP86/def2-TZVP

SCF-energy : -307.3097787
SCF + E(vib0) : -307.1453692

C 0.298498 -0.781679 -0.255172
N 0.173708 0.682329 -0.113845
C -0.921222 1.470299 -0.043887
N -2.129267 0.928338 0.224513
C -2.490755 -0.390661 0.779582
C 1.456347 1.371149 -0.243056
C -3.277290 1.825669 0.112855
H 1.264804 2.447555 -0.205179
H -4.010243 1.438110 -0.615230
H -0.629879 -1.214103 -0.637276
H 0.574038 -1.284076 0.687506
H 1.093611 -0.992392 -0.986317
H -2.910909 2.799318 -0.225532
H -3.787860 1.935001 1.086090
H 2.137368 1.091476 0.578664
H 1.948806 1.112364 -1.197435
H -3.292299 -0.246533 1.519906
H -2.867305 -1.091771 0.015872
H -1.637886 -0.841104 1.294333

I-Rh

BP86/def2-TZVP

SCF-energy : -1105.2426049
SCF + E(vib0) : -1105.0576593

C 0.133124 -0.824525 -0.452573
N 0.145036 0.622546 -0.222487
C -0.945423 1.414262 -0.153768
N -2.135561 0.867576 0.170793
C -2.308882 -0.285495 1.063317
C 1.481123 1.215333 -0.254980
C -3.364307 1.645533 -0.001774
H 1.390525 2.306970 -0.178096
H -4.145223 1.000283 -0.432803
H -0.850503 -1.146143 -0.806881
H 0.406631 -1.403901 0.443060
H 0.872327 -1.047154 -1.236175
H -3.170745 2.487971 -0.675982
H -3.707786 2.038771 0.967159
H 2.076492 0.847478 0.594773
H 1.994697 0.948784 -1.192546
H -3.011987 0.007230 1.856285
H -2.721397 -1.165026 0.545190
H -1.360442 -0.549330 1.538573
Rh -0.799055 3.495544 -0.413248
Cl -0.915553 3.561074 1.949421
C -0.687050 3.319794 -2.238442
C -0.685889 5.393155 -0.458070
O -0.618894 6.541947 -0.468242
O -0.608942 3.200032 -3.390757

II

BP86/def2-TZVP

SCF-energy : -306.1267456
SCF + E(vib0) : -305.9823047

C -0.007509 -0.412871 -0.171668
N 0.302049 1.015107 0.084267
C -0.772302 1.793820 0.324481
N -1.848368 0.993336 0.183579
C -1.541546 -0.395371 -0.238285
H 0.375128 -1.042603 0.650187
C 1.672338 1.448513 0.223251
C -3.217865 1.444570 0.258682
H -2.012000 -1.127221 0.436342
H -3.727627 1.355371 -0.717501
H -3.211646 2.496832 0.562145
H -3.791566 0.858007 0.996411
H 1.668243 2.524695 0.426312
H 2.179403 0.927893 1.055403
H 2.247174 1.256074 -0.698584
H -1.925303 -0.581738 -1.256465
H 0.461945 -0.758616 -1.105600

II-Rh

BP86/def2-TZVP

SCF-energy : -1104.0595059
SCF + E(vib0) : -1103.8944664

C -0.005204 -0.543047 0.081232
N 0.312360 0.894375 0.107049
C -0.783913 1.665957 0.088030
N -1.873549 0.880059 0.070688
C -1.536571 -0.543957 0.242638
C 1.682691 1.361620 0.090318
H 2.203991 1.014865 -0.816992
C -3.239263 1.339550 0.228891
H -3.260218 2.425238 0.075266
Rh -0.803160 3.736658 0.223128
Cl -0.893269 3.342930 2.547937
C -0.827860 5.611144 0.533348
C -0.734186 3.916856 -1.603594
O -0.843680 6.742943 0.739033
O -0.689956 4.015012 -2.759153
H 2.225163 0.993502 0.974677
H 1.673634 2.458145 0.110459
H -3.892693 0.849132 -0.508338
H -3.611876 1.121510 1.242877
H -2.048291 -1.161408 -0.509218
H -1.854305 -0.883905 1.242360
H 0.512322 -1.069768 0.895891
H 0.321001 -0.985220 -0.874938

III

BP86/def2-TZVP

SCF-energy : -304.9265539
SCF + E(vib0) : -304.8038871

C -0.093157 -0.392550 0.121579
N 0.291680 0.942112 0.200802
C -0.772529 1.807722 0.243769
N -1.838117 0.944558 0.187048
C -1.455337 -0.391009 0.112729
H 0.610261 -1.216868 0.078875
C 1.672864 1.395108 0.237288
C -3.218625 1.400772 0.204588
H -2.160046 -1.213723 0.060742
H -3.743027 1.098781 -0.713731
H -3.200336 2.493395 0.269619
H -3.755108 0.992327 1.073344
H 1.656244 2.488061 0.296822
H 2.195098 0.989640 1.116064
H 2.210386 1.087379 -0.671450

III-Rh

BP86/def2-TZVP

SCF-energy : -1102.8581136
SCF + E(vib0) : -1102.7153137

C -0.086804 -0.472207 0.131232
N 0.303774 0.858154 0.096245
C -0.777686 1.683559 0.116528
N -1.851728 0.848428 0.149468
C -1.447951 -0.478622 0.164321
C 1.687993 1.318333 0.110723
H 2.251048 0.841225 -0.701849
C -3.238193 1.295490 0.222418
H -3.241860 2.388594 0.138575
Rh -0.778497 3.755141 0.238483
Cl -0.588150 3.423224 2.565114
C -0.768133 5.631558 0.522625
C -0.919167 3.914975 -1.585825
O -0.760958 6.766099 0.712940
O -1.006723 4.003403 -2.739634
H 2.154248 1.085045 1.076785
H 1.681371 2.405858 -0.026558
H -3.820971 0.855597 -0.597478
H -3.677422 1.009544 1.186967
H 0.625760 -1.287823 0.130315
H -2.152247 -1.300715 0.196889

dipiy

BP86/def2-TZVP

SCF-energy : -533.6677968
SCF + E(vib0) : -533.5087582

C -0.077406 -0.454678 0.159361
N 0.286167 0.920443 0.234723
C -0.773025 1.801728 0.270550
N -1.833487 0.923059 0.214021
C -1.471924 -0.452954 0.145794
C 0.922295 -1.445729 0.116433
C 2.246993 -1.072627 0.148380
C 2.589614 0.313279 0.224696
C 1.623161 1.276103 0.266288
H 0.626975 -2.493566 0.058712
H 3.034059 -1.824731 0.116285
H 3.633987 0.620328 0.250956
H 1.808441 2.346077 0.324364
C -3.797192 -1.065121 0.089741
C -2.473052 -1.441522 0.083581
C -3.169954 1.282062 0.219471
C -4.137797 0.321650 0.159105
H -4.585359 -1.815277 0.042555
H -2.179286 -2.490114 0.031917
H -3.353654 2.352506 0.273789
H -5.181717 0.631287 0.164911

2a

BP86/def2-TZVP

SCF-energy : -1331.5791984
SCF + E(vib0) : -1331.4002511

C -0.064192 -0.463516 0.183608
N 0.319682 0.896906 0.208653
C -0.751286 1.752125 0.157114
N -1.831056 0.910968 0.103421
C -1.462876 -0.456256 0.115950
C 0.923597 -1.466249 0.224382
C 2.249727 -1.105533 0.296155
C 2.606931 0.274344 0.333931
C 1.655593 1.251840 0.292936
H 0.618205 -2.511729 0.200269
H 3.026683 -1.867869 0.330115
H 3.651832 0.571365 0.405472
H 1.846272 2.320803 0.345561
C -3.786169 -1.072156 0.010482
C -2.462385 -1.445924 0.065422
C -3.166978 1.278049 0.063828
C -4.129381 0.312265 0.012974
H -4.571192 -1.825776 -0.026779
H -2.168027 -2.494931 0.073989
H -3.349896 2.350161 0.088730
H -5.172966 0.621140 -0.017600
Rh -0.730136 3.822914 0.227203
Cl 0.156340 3.605852 2.401285
C -0.685466 5.706519 0.443897
C -1.348123 3.933966 -1.498613
O -0.659964 6.846660 0.595331
O -1.717215 4.002776 -2.596657

I-Rh

B3LYP/def2-TZVP

SCF-energy : -1104.6335491
SCF + E(vib0) : -1104.4432914

C 0.127405 -0.820257 -0.451815
N 0.138747 0.623196 -0.212633
C -0.946378 1.410979 -0.156040
N -2.130751 0.863785 0.159976
C -2.301348 -0.277688 1.064646
C 1.477679 1.204410 -0.250469
C -3.362644 1.634060 -0.009237
H 1.419059 2.274764 -0.073283
H -4.153003 0.966311 -0.360345
H -0.843421 -1.138582 -0.818968
H 0.385807 -1.399090 0.438607
H 0.870825 -1.038843 -1.221023
H -3.210426 2.421879 -0.741062
H -3.664873 2.085947 0.938661
H 2.093696 0.753536 0.531524
H 1.949460 1.017555 -1.220053
H -3.011766 0.013962 1.839790
H -2.693820 -1.161591 0.555550
H -1.364017 -0.523719 1.552681
Rh -0.801780 3.500888 -0.417979
Cl -0.934741 3.587053 1.958686
C -0.675906 3.323511 -2.251914
C -0.689479 5.417143 -0.457906
O -0.625155 6.551218 -0.456954
O -0.590853 3.202282 -3.388694

II-Rh

B3LYP/def2-TZVP

SCF-energy : -1103.4548907
SCF + E(vib0) : -1103.2852282

C -0.007060 -0.538876 0.085528
N 0.306867 0.894645 0.094076
C -0.783345 1.662008 0.076637
N -1.867604 0.880715 0.066457
C -1.534599 -0.538957 0.240121
C 1.678513 1.351450 0.086905
H 2.200662 0.992984 -0.805749
C -3.233676 1.331643 0.226804
H -3.277666 2.402340 0.042642
Rh -0.802904 3.742028 0.216693
Cl -0.890964 3.355264 2.558860
C -0.827726 5.632727 0.545339
C -0.737675 3.930417 -1.619744
O -0.843312 6.746601 0.765560
O -0.697323 4.030682 -2.760370
H 2.205248 0.986091 0.972316
H 1.688283 2.438644 0.098321
H -3.883447 0.813890 -0.482901
H -3.590071 1.138886 1.243127
H -2.039316 -1.152105 -0.508407
H -1.852938 -0.877112 1.231061
H 0.502845 -1.050334 0.903996
H 0.320375 -0.991440 -0.855681

III-Rh

B3LYP/def2-TZVP

SCF-energy : -1102.2568011
SCF + E(vib0) : -1102.1101720

C -0.090752 -0.464273 0.124669
N 0.297599 0.862149 0.078187
C -0.779221 1.679246 0.089848
N -1.846635 0.849995 0.123690
C -1.441497 -0.472696 0.151769
C 1.681317 1.314446 0.129732
H 2.297025 0.672270 -0.499322
C -3.235158 1.281066 0.199628
H -3.267271 2.358352 0.060744
Rh -0.769764 3.759138 0.234611
Cl -0.477578 3.399020 2.563444
C -0.741737 5.648352 0.556345
C -0.983106 3.947687 -1.589998
O -0.723357 6.763084 0.772006
O -1.112517 4.050849 -2.723712
H 2.046595 1.291961 1.156519
H 1.726434 2.337562 -0.233870
H -3.820628 0.791417 -0.578988
H -3.650152 1.038842 1.178313
H 0.617496 -1.273423 0.130662
H -2.139387 -1.290184 0.184008

2a

B3LYP/def2-TZVP

SCF-energy : -1330.8264350
SCF + E(vib0) : -1330.6424658

C -0.072144 -0.458179 0.181211
N 0.309268 0.894671 0.204824
C -0.756509 1.741897 0.154745
N -1.828752 0.905736 0.104870
C -1.459062 -0.452908 0.117062
C 0.916601 -1.460533 0.219104
C 2.230854 -1.101269 0.287783
C 2.588163 0.278525 0.326684
C 1.643968 1.247709 0.288815
H 0.613944 -2.498523 0.194689
H 3.004234 -1.856110 0.320292
H 3.626116 0.570906 0.398202
H 1.839873 2.306365 0.344082
C -3.769442 -1.077262 0.016349
C -2.456476 -1.445266 0.067753
C -3.164666 1.266812 0.068662
C -4.116799 0.306195 0.020632
H -4.548609 -1.825710 -0.019822
H -2.161447 -2.485831 0.074636
H -3.357018 2.328033 0.093913
H -5.154503 0.607296 -0.006048
Rh -0.731807 3.826287 0.226585
Cl 0.113112 3.620819 2.434734
C -0.674243 5.727398 0.447174
C -1.324841 3.934628 -1.519277
O -0.636289 6.851803 0.601398
O -1.675971 3.995226 -2.607991
